# Advancing Gastrointestinal Cancer Risk Prediction With Patient-Centered Machine Learning: Machine Learning Modeling Study

**DOI:** 10.2196/78931

**Published:** 2026-06-04

**Authors:** Daina Baublyte, Jeonghee Lee, Madhawa Gunathilake, Jeongseon Kim

**Affiliations:** 1 Department of Public Health & AI, National Cancer Center Graduate School of Cancer Science and Policy, National Cancer Center, Goyang-si, Republic of Korea; 2 Department of Cancer Biomedical Science, National Cancer Center Graduate School of Cancer Science and Policy, National Cancer Center, 323 Ilsan-ro, Ilsandong-gu, Gyeonggi-do, Goyang-si, 10408, Republic of Korea, 82 31-920-2570, 82 31-920-2579

**Keywords:** gastrointestinal cancer, class imbalance, machine learning, cohort study, data resampling, cancer risk prediction

## Abstract

**Background:**

Gastrointestinal (GI) cancers are a significant health concern in South Korea. Recently, machine learning (ML) models have emerged as powerful tools to support early screening efforts and identify people at risk before disease onset. However, the low incidence of GI malignancies in prospective cohorts leads to severe class imbalance, often causing ML models to favor the majority “healthy” class at the expense of clinical sensitivity.

**Objective:**

This study aimed to evaluate class imbalance mitigation strategies and develop ML-based GI cancer risk prediction models using noninvasive and minimally invasive predictors linked to modifiable behavioral and metabolic risk factors.

**Methods:**

We analyzed a prospective cohort (n=7652) with 156 incident GI cancer cases (2%) identified over a 14-year follow-up period. The data were randomly split into training (5356/7652, 70%) and testing (2296/7652, 30%) sets. To address class imbalance while preserving observed population structure, we developed a patient-centered undersampling technique (PCUSTe) based on the logic of frequency-matched case-control studies. PCUSTe was compared with commonly used resampling approaches, including synthetic minority oversampling (SMOTE), adaptive synthetic sampling (ADASYN), and SMOTE with edited nearest neighbors (ENN). Six classifiers were implemented, including both batch and incremental training variants. To account for the prior shift introduced by resampling, probability correction was applied. Model performance was evaluated on the independent test set using a classification threshold equal to the observed event proportion (cumulative incidence) in the training data and then across thresholds reflecting incidence values between 1% and 5%. Primary performance metrics included sensitivity, specificity, Matthews correlation coefficient, and area under the receiver operating characteristic curve (AUC).

**Results:**

Models trained using PCUSTe demonstrated improved sensitivity compared with standard resampling techniques, particularly for more complex classifiers. The incrementally trained stochastic gradient descent model achieved the highest overall performance trained on PCUSTe data with a sensitivity of 0.77 (95% CI 0.64‐0.89), specificity of 0.65 (95% CI 0.63‐0.67), AUC of 0.77 (95% CI 0.70‐0.84), and Matthews correlation coefficient of 0.12 (95% CI 0.08‐0.16). In contrast, logistic regression achieved balanced performance without resampling (sensitivity 0.70, 95% CI 0.57‐0.83; specificity 0.71, 95% CI 0.69‐0.72; AUC 0.75, 95% CI 0.68‐0.82). Our results showed that PCUSTe primarily enhanced sensitivity in more complex models at the expense of specificity.

**Conclusions:**

Integrating epidemiological principles, including covariate frequency matching and threshold selection based on the observed cumulative incidence in the training data, improved minority class detection in GI cancer risk prediction. However, model performance varied by algorithm, and in some cases, decision threshold adjustment alone achieved comparable or superior results to data resampling. These findings highlight the importance of carefully selecting imbalance mitigation strategies based on modeling objectives. The resulting models achieved sensitivity levels that may be suitable for early risk identification in cohort settings and could contribute to personalized risk stratification and targeted prevention or screening strategies.

## Introduction

Gastrointestinal (GI) cancers pose a significant global health burden, with nearly 5 million new cases and over 3 million deaths reported in 2022 [1-4]. The impact is particularly severe in Asia, where GI cancers account for nearly 30% of all cancer cases, largely due to lifestyle habits, dietary factors, *Helicobacter pylori* infections, and genetic predispositions [4-8]. These trends underscore the urgent need for region-specific prevention and early detection strategies.

Predictive models play a critical role in identifying high-risk individuals and enabling targeted preventive measures [9-11]. However, accurate risk prediction requires pre-diagnosis patient data, which is best obtained from the prospective cohort studies—longitudinal studies that track healthy individuals over extended periods. Compared with other observational designs, prospective cohorts are less susceptible to selection bias because they minimize reliance on recall and are robust to differential survival. Although they do not guarantee perfect generalizability, they typically provide a closer approximation of the true population. These studies offer high-quality, temporally structured data, but their high cost and resource-intensive nature limit their availability. Moreover, many potential risk factors involved in disease development exhibit complex, nonlinear relationships, posing additional challenges for traditional analytic approaches.

In this context, advances in machine learning (ML) offer a promising solution. With the increasing availability of large-scale health datasets, ML has emerged as a powerful tool for disease risk prediction, offering advantages over conventional statistical methods in modeling complex, multidimensional data [12,13]. ML-driven models can uncover nonlinear interactions among risk factors, improving predictive performance in early cancer detection. Although the application of ML methods has demonstrated promising results in GI cancer risk prediction using biomarkers, anthropometric measures, and socioeconomic data, its application remains limited [14,15].

One of the major challenges in applying ML to GI cancer risk prediction is the severe class imbalance inherent in cohort studies, where cancer cases constitute only a small fraction of the population. This imbalance skews model performance, leading to high accuracy for the majority class (noncancer) but poor sensitivity for cancer cases [16-18]. Additionally, integrating diverse risk factors—such as biomarkers, dietary intake, and lifestyle data—compounds this challenge, as these variables often exhibit high variability and sparse representation. Given the heterogeneous nature of GI cancers, incorporating a wider range of risk factors may support the development of more effective screening and prevention strategies [19-24].

While previous studies have been conducted for GI cancer risk prediction, many have relied on relatively balanced datasets from cross-sectional studies, narrow predictor sets, or traditional statistical models, which may not fully capture the complex, nonlinear interactions among diverse risk factors [12-15,25]. To address these limitations, this study used a diverse set of predictors and focused on risk prediction in highly imbalanced cohort data.

To address class imbalance in ML-based GI cancer risk prediction, we evaluated multiple imbalance mitigation strategies, including a patient-centered undersampling technique (PCUSTe) grounded in epidemiological principles of frequency-based case-control matching. PCUSTe was designed to preserve observed population structure in small, highly imbalanced cohorts and was compared with established resampling approaches, including synthetic minority oversampling (SMOTE), adaptive synthetic sampling (ADASYN), and hybrid resampling methods [26]. In addition to data-level strategies, we incorporated probability correction for models trained on resampled data and decision threshold adjustment based on observed cumulative incidence. Model behavior and performance were systematically compared across imbalance mitigation strategies using multiple ML algorithms and hyperparameter settings to examine their influence on GI cancer risk prediction in highly imbalanced cohort data.

## Methods

### Study Design and Participants

**Figure 1.**
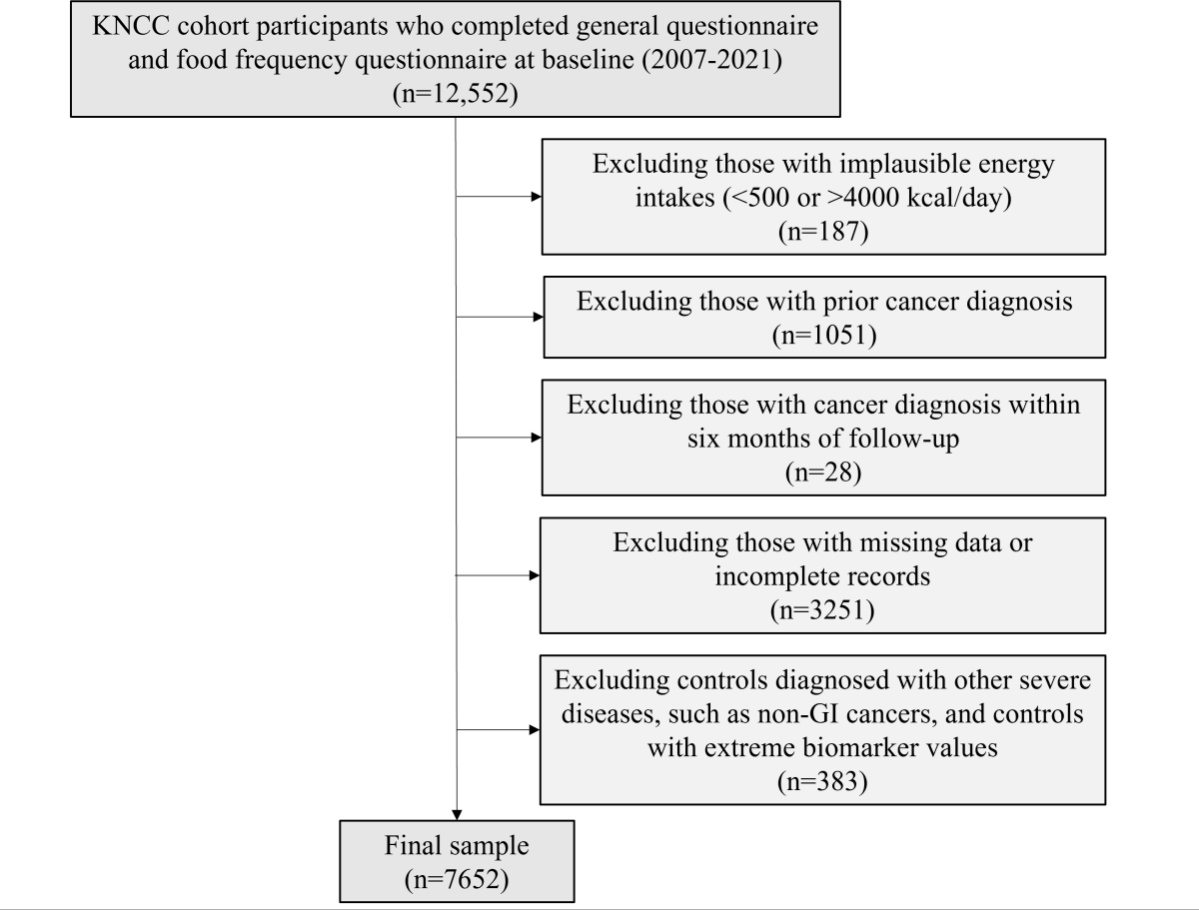
Flowchart of the study participant selection. GI: gastrointestinal; KNCC: Korea National Cancer Center.

This study used data from 12,552 South Korean adults enrolled in the Korea National Cancer Center (KNCC) Screenee Cohort, a longitudinal study initiated in 2002 by the National Cancer Center in South Korea [27]. To ensure data quality and minimize potential bias, several exclusion criteria were applied. Participants were excluded if they had a prior cancer diagnosis (n=1051), were diagnosed with GI cancer within 6 months of follow-up (n=28), or reported implausible total energy intake (<500 or >4000 kcal/day), which may indicate dietary misreporting or data entry errors (n=187). Participants with missing or incomplete records were also excluded, and a complete-case analysis was conducted (n=3251). In addition, controls with other severe conditions—including non-GI cancers or extreme biomarker values suggestive of acute metabolic or hepatic abnormalities—were excluded. These abnormalities were defined as triglycerides, aspartate aminotransferase, or gamma-glutamyl transferase levels exceeding 1000, or fasting glucose levels below 55 or above 200 mg/dL (n=383) [28-30]. Participant selection and exclusion steps are illustrated in Figure 1.

Participants were followed for up to 14 years (2007‐2021). GI cancers were defined according to the *ICD-10* (*International Statistical Classification of Diseases, Tenth Revision*) as follows: esophageal cancer (C15), gastric cancer (C16), small intestine cancer (C17), colorectal cancer (C18-C20), anal cancer (C21), liver cancer (C22), gallbladder cancer (C23-C24), and pancreatic cancer (C25). Individuals who developed GI cancer during the follow-up period were classified as cases, while those who remained cancer-free served as controls. Based on this labeling, a supervised ML framework was constructed to address a binary classification task—identifying individuals at risk for developing GI cancer.

The final dataset consisted of 7652 participants (61% of the original cohort), including 156 (2.0%) incident GI cancer cases and 7496 (98.0%) controls. Some participants developed malignancies at multiple GI sites, resulting in a total of 162 cancer diagnoses. The most frequently diagnosed cancers were gastric (62/162, 38.3%), colorectal (43/162, 26.5%), and hepatic (30/162, 18.5%).

### Data Collection and Preprocessing

All participants in the KNCC Screenee Cohort completed self-administered questionnaires capturing socio-demographic characteristics and lifestyle behaviors. Additionally, data were collected on anthropometric measurements, clinical biomarkers, and dietary intake. Dietary intake was assessed at baseline using a validated 106-item semiquantitative food frequency questionnaire [31,32].

To adjust for total energy intake’s influence on nutrient intake, we applied the residual method in Python (version 3.12) [33]. Categorical variables, such as lifestyle habits, gender, and socioeconomic characteristics, were binarized, and low-density lipoproteins were calculated using Friedewald’s equation [34].

The dataset was randomly split into a training set (5356/7652, 70%) and a test set (2296/7652, 30%). The training set included 109 cases and 5247 controls, while the test set consisted of 47 cases and 2249 controls. Continuous variables were standardized using an adapted Z-score normalization based on the control population. For each predictor, the mean and standard deviation were calculated using only control individuals in the training set, and these parameters were applied to transform both cases and controls in the training data. The same parameters were then applied to the corresponding test data.

### Ethical Considerations

This study was conducted as a secondary analysis of existing cohort data from participants who had previously provided broad informed consent for future research use at enrollment in the KNCC Screenee Cohort. The study was noninterventional and did not affect participant care. Personal identifiers were used solely for pseudonymized linkage with national cancer registry data by an authorized data linkage institution and were removed immediately thereafter. Only deidentified data were accessed by authorized research personnel within a secure analysis environment. Given the use of deidentified data and the absence of participant contact or intervention, the risk to participants was considered minimal, and no additional informed consent, compensation, or participant support was required. This study was reviewed and approved by the Institutional Review Board of the National Cancer Center, Korea (approval number NCC2024-0106).

### Statistical Analysis

Descriptive statistics were generated using Python (version 3.12) following the energy adjustment of nutrient intakes. To ensure the representativeness of the study population and identify potential selection or partitioning bias, baseline characteristics were compared across several cohorts: the final analyzed cohort, individuals excluded due to missing data, and the training and test data splits. Continuous variables were summarized as means and standard deviations and evaluated using Welch *t* test to account for potential unequal variances. Categorical variables were expressed as frequencies and percentages, with differences assessed using Pearson chi-square tests [35]. All statistical tests were 2-sided, and a *P* value <.05 was considered to indicate statistical significance.

### Sample Size Considerations

In the absence of a universally accepted criterion for determining sample size adequacy in ML prediction models, we adopted the event-per-variable (EPV) framework from regression modeling as a conservative reference. Although an EPV of ≥10 has traditionally been recommended for logistic regression (LR), recent evidence suggests that lower EPV values may be acceptable when penalization and shrinkage techniques are applied [36]. With 109 GI cancer cases and 20 selected predictors, the EPV for tuned models in our study was 5.45:


$$EPV=\frac{Number\;of\;Events}{Number\;of\;Predictors}=\frac{109}{20}=5.45$$

In addition to tuned configurations, we evaluated baseline models using all 34 available predictors without hyperparameter tuning or feature selection. This approach was adopted to examine whether systematic tuning materially altered predictive performance, given that default parameter settings may perform comparably to, or occasionally outperform, extensively tuned models. In these baseline configurations, the nominal EPV decreased below the selected threshold.

All evaluated models incorporated model-specific regularization or complexity control mechanisms. To further assess robustness, we examined model stability across a range of EPV values from 9.9 (11 predictors) to 3.2 (34 predictors). These analyses were conducted to evaluate stability across model complexities rather than to infer optimal predictive performance.

### Data Resampling Strategies

#### Algorithm Selection

Resampling methods are commonly used to address class imbalance and can be broadly categorized as oversampling or undersampling. Oversampling enlarges the minority class by generating synthetic observations, which may improve sensitivity but can introduce synthetic noise and overfitting. Undersampling, in contrast, reduces the majority class size to preserve data authenticity but risks discarding informative samples and distorting population structure. To achieve a better balance between data integrity and population representativeness, we developed PCUSTe, a patient-centered undersampling method.

#### Patient-Centered Undersampling Method

Similar to other undersampling methods, PCUSTe addresses class imbalance without generating synthetic data. However, unlike conventional methods that randomly remove majority-class samples or rely on geometric distances in feature space, PCUSTe uses case distribution guided sampling based on categorical matching covariates. Controls are selected in proportion to the empirical distribution of cases across these strata, ensuring that the resulting training data preserve the covariate composition of the study population. This approach integrates the design logic of frequency-matched case-control studies into ML preprocessing context, thereby minimizing distributional bias introduced by random or distance-based undersampling.

The method is parameterized by the case-to-control ratio (1:1 in this study, though extendable) and the covariates guiding sampling. Random seeds can be specified to ensure reproducibility. Finally, the combined dataset is shuffled to prevent ordering bias. In this study, we tested 2 parameterizations: PCUSTe-1 (PCUSTe with sociodemographic matching [education, employment, income, and marital status]) and PCUSTe-2 (PCUSTe with lifestyle matching [smoking and drinking]). The pseudocode for the PCUSTe method is provided in the Multimedia Appendix 1.

#### Synthetic Oversampling Methods

To complement the undersampling strategy, we also implemented synthetic oversampling using 3 established methods. SMOTE generates new samples of the minority class by interpolating between existing cases. ADASYN extends this approach by focusing more on generating samples near harder-to-classify instances. Additionally, we used a hybrid technique that combines SMOTE with edited nearest neighbors (ENN), which removes misclassified samples following oversampling to sharpen class boundaries and improve data quality.

We further optimized SMOTE, SMOTE+ ENN, and ADASYN by varying the k-nearest neighbors (k-NN) parameter as a function of the training set size. Specifically, k was defined as a proportion of the number of cases in the training data, and 6 values were evaluated: 2% (2/109), 5% (5/109), 10% (10/109), 30% (32/109), 50% (54/109), and 80% (87/109) of the case count. For reference, setting k to 5% of n resulted in 5 neighbors, which is the default setting for all 3 methods.

### ML Modeling

#### Algorithm Selection

We implemented a range of ML algorithms selected for their relevance to cancer risk prediction. LR served as the baseline model due to its simplicity, high interpretability, and computational efficiency, and was implemented with regularization to improve generalizability [37]. To explore the difference between batch and incremental learning approaches, we also applied stochastic gradient descent (SGD), which represents an incremental version of LR suitable for streaming or large-scale data applications [38]. In addition to linear models, we used random forest (RF), a decision-tree-based ensemble algorithm known for its robustness and ability to model nonlinear relationships [39].

To assess the benefits of more sophisticated models, we implemented extreme gradient boosting (XGBoost), which is widely recognized for its performance in structured data and its ability to handle class imbalance effectively [40]. Both batch and incremental training modes were explored for XGBoost. Support vector machines (SVM) were included as well, given their efficacy in handling high-dimensional datasets and their resilience to overfitting in small, imbalanced datasets [41].

To maximize the utility of the PCUSTe, we implemented an incremental learning variant using the SGD classifier. This approach involved 1000 training iterations, where each iteration generated a unique undersampled training set maintaining the specified covariate matching constraints. The iterative strategy was designed to mitigate the primary drawback of undersampling—the loss of information—by ensuring the model was exposed to more of the control population over time. For reproducibility, the random seed for each PCUSTe generation was updated by incrementing a base seed by the current iteration index. While the case samples remained constant across all iterations, the control subsets varied, allowing for partial overlap and a more robust representation of the original control distribution than a single-pass undersampling would allow. This high number of iterations was selected to ensure that the vast majority of the control population was used in the optimization process.

#### Predictor Selection

To ensure methodological consistency across resampling strategies, and because SMOTE-based approaches generate synthetic observations via interpolation in continuous feature space, predictor selection was restricted to continuous variables. Recursive feature elimination was applied using each target model’s own baseline estimator as the base learner, rather than a shared estimator across models.

Predictor selection was conducted exclusively within the training data to prevent information leakage and was integrated into the cross-validation pipeline together with hyperparameter optimization. To evaluate predictor relevance and stability across configurations, feature importance was further examined using Shapley Additive Explanations (SHAP) analysis [42].

#### Hyperparameter Optimization

Hyperparameter optimization was performed using RandomizedSearchCV (RS) exclusively on training data. Two performance metrics were used as optimization objectives: area under the receiver operating characteristic curve (AUC) and Matthews correlation coefficient (MCC). This resulted in 3 parameter configurations per model: None (default model settings), RS-AUC, and RS-MCC. The dual-objective optimization strategy allowed us to evaluate both probabilistic discrimination (AUC) and balanced classification performance (MCC), which is particularly relevant in imbalanced settings.

For SGD models, hyperparameter tuning was constrained to preserve incremental training properties: L2 regularization was fixed a priori, tolerance was disabled, and the number of iterations was set to one. Consequently, RS was applied only to selected learning-rate and regularization strength parameters (eta0 and alpha).

Tuned model development was implemented within a unified k-fold cross-validation pipeline. In each iteration, k–1 folds were used for training and hold-out fold for validation. Resampling was applied exclusively to the training folds, followed by recursive feature elimination for feature selection and RS for hyperparameter optimization. Performance was evaluated on the held-out validation fold. The independent test set was not involved in any stage of model development.

#### Performance Evaluation

To comprehensively assess model performance, evaluation metrics specifically suited for imbalanced data were prioritized [43,44]. Sensitivity, AUC, MCC, and specificity served as primary metrics to capture class-wise discrimination and model separability. Secondary metrics, including positive predictive value, negative predictive value, Brier score, accuracy, and overall weighted *F*_1_-scores, were also estimated to provide a complete view of precision and calibration. Performance metrics were computed with 95% CIs estimated using 1000 bootstrap iterations.

Because resampling alters the class prior and can distort predicted probabilities, models trained on resampled data underwent prior probability correction to restore calibration relative to the original class distribution [45]. Decision-based performance metrics (sensitivity, specificity, and MCC) were then computed using a classification threshold of 0.02, corresponding to the observed disease incidence in the training cohort. This threshold reflects a deployment-aligned operating point, where individuals with predicted risk exceeding the baseline population incidence would be considered high risk. Thresholds were defined to reflect epidemiologically plausible deployment scenarios, where the decision cutoff may be aligned with a priori expected disease incidence rather than determined by maximizing performance metrics within the training data. Sensitivity analyses were conducted across thresholds ranging from 0.01 to 0.05 to assess robustness under plausible real-world incidence scenarios.

After model development, all configurations (crude and tuned) were evaluated once on the reserved independent test set without further modification. The test set served as a common hold-out benchmark for comparing predefined algorithm–resampling–tuning configurations. For descriptive comparison, one configuration per ML algorithm was additionally evaluated against a null incidence-based model defined as a classifier that predicts the minority class for all observations. Under this specification, the baseline represents the upper bound for recall and the lower bound for precision given the observed outcome incidence.

To differentiate between generalization, learning, and memorization, model performance was compared across the original imbalanced test set, the original imbalanced training set, and the resampled training datasets. This framework enabled direct assessment of the models’ ability to distinguish classes while facilitating detection of potential overfitting to synthetic noise or oversampling artifacts.

### Data Distribution Analysis

To assess how resampling techniques influenced data distribution, principal component analysis (PCA) was used for 2D projection and visual inspection of structural shifts. PCA reduces high-dimensional data into orthogonal components that capture the greatest proportion of variance, enabling visual comparison of the structural characteristics of the original and resampled datasets [46].

To quantify overall separation between outcome groups, the mean Euclidean distance between cases and controls was calculated for each dataset. Pairwise Euclidean distances were computed between all case and control observations in the standardized feature space, and the average of these distances was used as a summary measure of inter-group separation.

In addition, predictor-level associations with the outcome were examined by calculating correlations between each individual predictor and the binary outcome variable. Correlation coefficients were computed independently for each dataset.

## Results

### Study Participant Characteristics

The study identified significant demographic, clinical, and dietary differences between cases and controls. GI cancer cases were more likely to be male, older, smokers, and to have a lower monthly income (below 2 million Korean won) compared with controls, while no significant differences were observed for alcohol consumption, marital status, employment, or education. Clinically, cases showed significantly higher BMI, systolic blood pressure (SBP), diastolic blood pressure (DBP), fasting blood glucose, aspartate aminotransferase, and gamma-glutamyl transferase, along with lower high-density lipoprotein cholesterol. Dietary intake differed modestly, with cases reporting significantly lower intakes of sugar, dietary fiber, niacin, potassium, and vitamin C, whereas total energy intake and most other nutrients did not differ significantly between groups. Detailed statistics of the whole study population are presented in Table 1.

**Table 1.** Baseline characteristics of the analyzed cohort.

Characteristics	Case (n=156)	Control (n=7496)	*P* value
Sex, n (%)			<.001
Male	89 (57.1)	2690 (35.9)	
Female	67 (42.9)	4806 (64.1)	
Alcohol consumer, n (%)			.68
No	55 (35.3)	2790 (37.2)	
Yes	101 (64.7)	4706 (62.8)	
Smoker, n (%)			<.001
No	79 (50.6)	5003 (66.7)	
Yes	77 (49.4)	2493 (33.3)	
Married or cohabitating, n (%)			>.99
No	19 (12.2)	901 (12.0)	
Yes	137 (87.8)	6595 (88.0)	
Unemployed, n (%)			.77
No	92 (59.0)	4307 (57.5)	
Yes	64 (41.0)	3189 (42.5)	
Higher education^a^, n (%)			.18
No	92 (59.0)	3994 (53.3)	
Yes	64 (41.0)	3502 (46.7)	
Lower income^b^, n (%)			.001
No	108 (69.2)	6009 (80.2)	
Yes	48 (30.8)	1487 (19.8)	
Age (years), mean (SD)	57.51 (7.83)	52.33 (8.20)	<.001
AST^c^ (U/L), mean (SD)	28.58 (15.06)	22.97 (11.11)	<.001
BMI (kg/m^2^), mean (SD)	24.97 (2.98)	23.59 (2.97)	<.001
Calcium (mg/d), mean (SD)	474.73 (199.97)	486.88 (211.73)	.45
Carbohydrate (g/d), mean (SD)	315.48 (33.92)	314.08 (33.71)	.61
β-Carotene (㎍/d), mean (SD)	1976.90 (1024.43)	2127.04 (1221.98)	.07
Cholesterol (mg/d), mean (SD)	121.09 (74.57)	131.70 (77.08)	.09
DBP^d^ (mmHg), mean (SD)	81.81 (10.82)	76.03 (10.48)	<.001
Fasting blood glucose (mg/dL), mean (SD)	102.44 (33.66)	92.99 (14.18)	<.001
Fat (g/d), mean (SD)	29.18 (11.78)	30.46 (11.53)	.18
Fiber (g/d), mean (SD)	15.13 (6.61)	16.69 (7.47)	.004
GGT^e^ (IU/L), mean (SD)	44.47 (68.35)	28.79 (33.74)	.005
HDL^f^ (mg/dL), mean (SD)	55.51 (13.01)	59.76 (14.59)	<.001
Iron (mg/d), mean (SD)	10.34 (2.60)	10.34 (2.88)	.97
LDL^g^ (mg/dL), mean (SD)	114.15 (36.13)	116.95 (32.64)	.34
Magnesium (mg/d), mean (SD)	168.12 (65.97)	177.43 (67.89)	.08
MUFA^h^ (g/d), mean (SD)	7.48 (3.74)	8.04 (3.93)	.06
Niacin (mg/d), mean (SD)	9.80 (2.42)	10.31 (2.59)	.01
Phosphorus (mg/d), mean (SD)	874.99 (206.04)	899.72 (221.95)	.14
Potassium (mg/d), mean (SD)	2319.55 (727.02)	2460.60 (800.13)	.03
Protein (g/d), mean (SD)	61.88 (10.73)	62.49 (11.41)	.48
PUFA^i^ (g/d), mean (SD)	4.60 (2.04)	4.84 (2.06)	.14
SBP^j^ (mmHg), mean (SD)	131.74 (15.07)	124.72 (14.46)	<.001
SFA^k^ (g/d), mean (SD)	8.45 (4.04)	9.04 (4.27)	.08
Sodium (mg/d), mean (SD)	1990.97 (814.69)	1981.94 (778.54)	.89
Sugar intake (g/d), mean (SD)	44.68 (25.30)	51.35 (28.39)	.001
Thiamin (mg/d), mean (SD)	0.90 (0.23)	0.92 (0.23)	.33
Energy (kcal/d), mean (SD)	1778.40 (597.48)	1732.69 (577.23)	.35
Triglyceride (mg/dL), mean (SD)	120.69 (65.40)	117.50 (75.34)	.55
Vitamin A (㎍ RE/d), mean (SD)	419.72 (201.31)	449.03 (224.47)	.07
Vitamin C (mg/d), mean (SD)	60.98 (34.46)	68.47 (39.26)	.008
Vitamin D (㎍/d), mean (SD)	4.37 (4.19)	4.79 (4.07)	.21
Vitamin E (mg/d), mean (SD)	6.34 (3.32)	6.65 (3.11)	.22
Zinc (mg/d), mean (SD)	4.93 (1.77)	5.16 (1.88)	.14

a Higher education: college or above.

b Lower income: below 2 million Korean won per month.

c AST: aspartate aminotransferase.

d DBP: diastolic blood pressure.

e GGT: gamma-glutamyl transferase.

f HDL: high-density lipoprotein.

g LDL: low-density lipoprotein.

h MUFA: monounsaturated fatty acids.

i PUFA: polyunsaturated fatty acids.

j SBP: systolic blood pressure.

k SFA: saturated fatty acids.

A comparative analysis of baseline characteristics revealed no critical concerns regarding the representativeness of the data splits or the impact of participant exclusion (Tables S1-S4 in Multimedia Appendix 2). Characteristics of the training and test datasets were highly consistent, although minor discrepancies in statistical significance were observed for some nutritional variables, including total fat, zinc, thiamin and vitamin D, and fatty acids. However, these discrepancies were primarily driven by the differences in statistical power between the 2 partitions, as the absolute mean values and standard deviations remained stable across both sets.

Similarly, participants excluded due to missing data exhibited baseline characteristics generally consistent with those of the analyzed cohort. While the magnitude of statistical significance varied between these groups, the underlying clinical trends remained stable across all comparisons. For instance, although the association between drinking status and the outcome only reached statistical significance within the excluded cohort, cases were more likely to report alcohol consumption in both groups. Likewise, mean BMI was consistently higher among cases in both cohorts, though this difference only attained statistical significance in the larger analyzed cohort. Collectively, these findings suggest that no critical systematic differences exist between the analyzed, excluded, and partitioned groups, indicating that neither inclusion bias nor data-splitting artifacts materially affected the study results.

### Data Resampling Strategies

#### Patient-Centered Undersampling Method

PCUSTe enabled stronger performance for nonlinear models. However, resampling approaches were less effective for linear models. Notably, adjusting the threshold for model probabilities was sufficient to stabilize LR performance and achieve a 0.7 score for both sensitivity and specificity on crude, nonresampled data. Similarly, RF performed well while trained on crude training data, although results were improved after model tuning and PCUSTe resampling.

#### Synthetic Oversampling Methods

**Figure 2.**
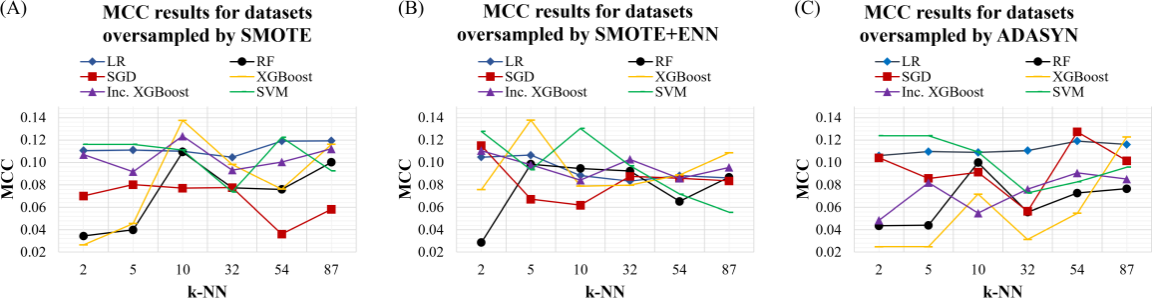
MCC scores of models evaluated on datasets oversampled using 3 different techniques: (A) SMOTE (left); (B) SMOTE+ ENN (middle); (C) ADASYN (right). Each subplot shows performance across varying values of the k parameter (number of nearest neighbors used for resampling): 2% (2/109), 5% (5/109), 10% (10/109), 30% (32/109), 50% (54/109), and 80% (87/109). Lines represent individual models, including logistic regression (LR), random forest (RF), stochastic gradient descent (SGD), support vector machine (SVM), and 2 extreme gradient boosting (XGBoost) variants. ADASYN: adaptive synthetic sampling; ENN: edited nearest neighbors; k-NN: k-nearest neighbors; LR: logistic regression; MCC: Matthews correlation coefficient; RF: random forest; SVM: support vector machine; XGBoost: extreme gradient boosting.

Experiments evaluating k-NN parameter tuning for synthetic oversampling methods indicated that the default k-NN setting may not be optimal and that tuning this parameter can substantially influence model performance. In our study, higher k-NN values—corresponding to approximately 10%‐80% of the minority class sample size—often yielded better performance than the default setting. These findings suggest that, when applying synthetic oversampling to small and extremely imbalanced datasets, tuning the k-NN parameter should be explicitly considered. We present an example of model performance based on MCC metric for each ML algorithm trained on oversampled data with different k-NN in Figure 2 (all models with default parameter settings).

### ML Modeling

Tuned model hyperparameters, feature sets, and optimization metric scores for the selected configuration are presented in Tables S5 and S6 in Multimedia Appendix 2.

#### Predictor Selection

SHAP analyses revealed substantial algorithm-specific differences in dominant predictors, with linear and incrementally trained models emphasizing dietary exposures, whereas tree-based models prioritized clinical and physiological markers (Figure S1, in Multimedia Appendix 2). Across all models, higher age consistently emerged as an important predictor of GI cancer risk; however, in the LR and SGD models, its relative influence was lower than that of dietary factors, including macronutrients, saturated fatty acids, and selected micronutrients. Elevated blood pressure was another consistently influential predictor across modeling approaches.

EPV sensitivity analysis results are presented in Figure S2 in Multimedia Appendix 2. As the number of predictors decreased (resulting in higher EPV ratios), MCC scores generally tended to decline across most models, although the magnitude of change varied by algorithm. In this study, the number of predictors was predefined (34 for crude models and 20 for tuned models). These findings suggest that reducing the predictor set may lead to information loss and that model performance could potentially be improved through more advanced predictor selection approaches.

#### Hyperparameter Optimization

RS yielded distinct optimal hyperparameter configurations depending on both the resampling strategy and the optimization metric. Based on inner cross-validation results within the training data, the highest AUC scores for LR, RF, and XGBoost models were achieved with PCUSTe-1 resampling, whereas SVM achieved the highest cross-validation AUC with SMOTE resampling. In contrast, MCC values were more consistent across resampling strategies, with most models achieving similar performance regardless of the sampling method used.

SVM models demonstrated metric-dependent kernel selection. Linear kernels were predominantly selected when optimizing for AUC, whereas radial basis function kernels were favored when optimizing for MCC in models trained on oversampled data. Under PCUSTe variants and hybrid resampling strategies, linear kernels were selected across both optimization metrics.

#### Performance Evaluation

##### Overall Performance

Despite pronounced class imbalance, some models trained on crude (nonresampled) data achieved moderate discriminatory performance when decision thresholds were set to the empirical disease incidence in training data. Under this setting, linear classifiers exhibited relatively stable behavior. Notably, the LR model trained on the original (nonresampled) data achieved the best performance among all algorithms in the crude evaluation. Importantly, without threshold adjustment, this model predicted all test set samples as controls. This finding suggests that when a strong linear signal is present, resampling may offer limited benefit, particularly when balanced sensitivity-specificity trade-offs are prioritized over aggressive minority class detection.

Undersampling methods consistently improved sensitivity across all model types, aligning better with screening-oriented objectives and the purpose of the classification task of our study. In contrast, oversampling approaches generally increased specificity at the expense of sensitivity, indicating a tendency to favor the majority class. Nonlinear models benefited most from undersampling strategies, whereas the same models trained on crude or oversampled data exhibited performance patterns consistent with majority-class overfitting. Detailed results for crude model evaluations are provided in Table S7 in Multimedia Appendix 2.

Tuned model performance results are reported in Tables S8 and S9 in Multimedia Appendix 2. Performance differences across resampling strategies were marginal for linear models but more pronounced for nonlinear models. Across most comparisons, PCUSTe-based methods outperformed oversampling approaches.

Table 2 contrasts one model configuration for each ML algorithm with the null (incidence-only) model, which classifies all samples as belonging to the minority class. All results are reported for the unseen nonresampled test set, the nonresampled training set, and the resampled training set. The selected configurations were not modified after the initial evaluation on the test set, ensuring that the results reflect predefined configurations and enabling comparison of model performance across ML algorithms and potential overfitting patterns.

**Table 2.** Performance results by machine learning algorithms on the original test and train, and resampled train datasets. Values represent point estimates with 95% CIs calculated by bootstrapping.

Model	Point estimate (95% CI)			
	Sensitivity	Specificity	AUC^a^	MCC^b^
Null^c^				
Test set	1.00	0.00	0.50	0.00
LR^d^				
Test set	0.70 (0.57‐0.83)	0.71 (0.69‐0.72)	0.75 (0.68‐0.82)	0.13 (0.08‐0.17)
Train set (original)	0.71 (0.62‐0.79)	0.70 (0.68‐0.71)	0.78 (0.73‐0.82)	0.12 (0.09‐0.15)
SGD^e^				
Test set	0.77 (0.64‐0.89)	0.65 (0.63‐0.67)	0.77 (0.70‐0.84)	0.12 (0.08‐0.16)
Train set (original)	0.77 (0.69‐0.85)	0.63 (0.61‐0.64)	0.78 (0.73‐0.81)	0.12 (0.09‐0.14)
Train set (resampled)	0.77 (0.69‐0.85)	0.61 (0.53‐0.70)	0.78 (0.72‐0.84)	0.39 (0.27‐0.50)
RF^f^				
Test set	0.77 (0.65‐0.89)	0.62 (0.60‐0.64)	0.73 (0.65‐0.81)	0.11 (0.07‐0.15)
Train set (original)	1.00 (1.00‐1.00)	0.62 (0.61‐0.63)	1.00 (1.00‐1.00)	0.18 (0.16‐0.20)
Train set (resampled)	1.00 (1.00‐1.00)	1.00 (1.00‐1.00)	1.00 (1.00‐1.00)	1.00 (1.00‐1.00)
XGB^g^				
Test set	0.77 (0.65‐0.89)	0.60 (0.58‐0.62)	0.73 (0.66‐0.79)	0.11 (0.07‐0.14)
Train set (original)	0.95 (0.91‐0.99)	0.59 (0.58‐0.60)	0.83 (0.81‐0.86)	0.16 (0.14‐0.17)
Train set (resampled)	0.99 (0.98‐0.99)	0.82 (0.80‐0.83)	0.98 (0.97‐0.98)	0.84 (0.83‐0.86)
Inc. XGB^h^				
Test set	0.70 (0.57‐0.83)	0.62 (0.60‐0.64)	0.68 (0.61‐0.76)	0.09 (0.05‐0.13)
Train set (original)	1.00 (1.00‐1.00)	0.62 (0.60‐0.63)	0.96 (0.95‐0.96)	0.18 (0.16‐0.19)
Train set (resampled)	1.00 (1.00‐1.00)	1.00 (1.00‐1.00)	1.00 (1.00‐1.00)	1.00 (1.00‐1.00)
SVM^i^				
Test set	0.79 (0.67‐0.90)	0.60 (0.58‐0.62)	0.74 (0.67‐0.80)	0.11 (0.07‐0.15)
Train set (original)	0.80 (0.72‐0.88)	0.59 (0.58‐0.60)	0.80 (0.75‐0.84)	0.11 (0.09‐0.14)
Train set (resampled)	0.80 (0.72‐0.87)	0.80 (0.72‐0.87)	0.87 (0.83‐0.91)	0.60 (0.49‐0.69)

a AUC: area under the receiver operating characteristic curve.

b MCC: Matthews correlation coefficient.

c Null: model that always predicts positive class (incidence-only).

d LR: logistic regression trained on nonresampled training split, crude model.

e SGD: stochastic gradient descent trained incrementally on varying data resampled by PCUSTe-1 method, crude model.

f RF: random forest trained on data resampled by PCUSTe-2 method, crude model.

g XGBoost: extreme gradient boosting trained on data resampled by SMOTE+ ENN (k=54) method, tuned via RS-AUC.

h Inc. XGBoost: extreme gradient boosting trained on data resampled by PCUSTe-2 method, crude model.

i SVM: support vector machine trained on data resampled by PCUSTe-2 method, crude model.

**Figure 3.**
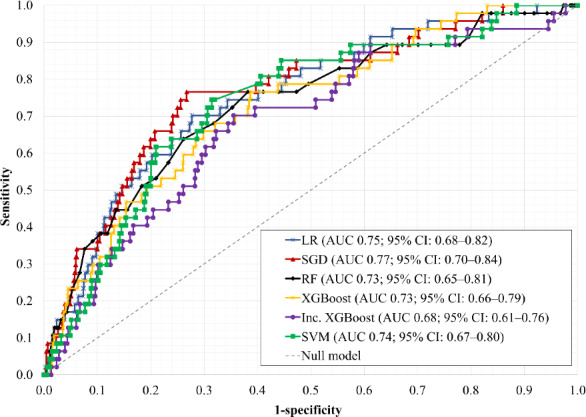
ROC curves for the selected configuration of each ML algorithm. AUC values in the legend represent point estimates on the test set with 95% CIs calculated by bootstrapping. AUC: area under the receiver operating characteristic curve; LR: logistic regression; RF: random forest; ROC: receiver operating characteristic; SGD: stochastic gradient descent; SVM: support vector machine; XGBoost: extreme gradient boosting.

Although MCC values were numerically low (0.09‐0.18) for the test and original training datasets, this reflects the extreme class imbalance in these sets, where even a small number of false-positive predictions disproportionately reduces MCC. Because the resampled training set was balanced across classes, MCC values appear substantially higher in that dataset. To better contextualize predictive performance, MCC values can be compared with those of the null model (MCC=0). Based on this comparison, the developed models achieved improvements of up to 0.13 in MCC, which may represent meaningful discrimination given the very low disease incidence in the test set. Overall, all ML algorithms demonstrated reasonable performance under at least one configuration. Receiver operating characteristic–AUC curves for selected model configurations are shown in Figure 3, and secondary performance metrics are provided in Table S10 in Multimedia Appendix 2. Compared with the null model, the developed models improved positive predictive value from 0.02 to up to 0.05 and achieved a high negative predictive value (up to 0.99), while maintaining a similar Brier score (0.02).

##### LR Model

During model evaluation, the LR model trained on resampled datasets demonstrated a modest increase in sensitivity compared with the model trained on the original nonresampled data, and notably degraded specificity. Thus, for ML algorithm-based comparison, we have retained the crude LR model with all predictors and default hyperparameters. This model demonstrated balanced performance, achieving a test sensitivity of 0.70 (95% CI 0.57‐0.83), specificity of 0.71 (95% CI 0.69‐0.72), and an AUC of 0.75 (95% CI 0.68‐0.82). Importantly, the model showed no evidence of overfitting, with performance metrics remaining consistent across data splits.

##### SGD Model

SGD, particularly when trained using the incrementally implemented PCUSTe-1 strategy with no tuning, achieved the strongest overall performance among the evaluated models. The highest observed performance was sensitivity of 0.77 (95% CI 0.64‐0.89), specificity of 0.65 (95% CI 0.63‐0.67), AUC of 0.77 (95% CI 0.70‐0.84), and MCC of 0.12 (95% CI 0.08‐0.16). Performance on training sets was highly consistent, although slightly lower compared with the test set. This model misclassified 11 out of 47 cancer cases as controls. Among the false negatives, diagnoses included colorectal (6/11), gastric (3/11), liver (1/11), and esophageal cancer (1/11), indicating that misclassification was not confined to a single GI cancer subtype.

##### RF Model

RF showed over-optimization to the resampled training distribution, with a perfect training AUC that decreased significantly on the test set 0.73 (95% CI 0.65‐0.81). Nevertheless, the model maintained a test sensitivity of 0.77 (95% CI 0.65‐0.89) and specificity of 0.62 (95% CI 0.60‐0.64) while trained on PCUSTe-2 dataset, suggesting moderate generalization despite evidence of overfitting. Notably, RF exhibited majority class overfitting when trained on all oversampling-based datasets. In contrast, no resampling or undersampling via PCUSTe resulted in a more balanced classification.

##### XGBoost Model

Boosting models exhibited high sensitivity to hyperparameter tuning, especially when trained on oversampled data. The batch-trained XGBoost model demonstrated strong predictive capacity but exhibited substantial performance variability between training and test environments. Although it achieved a test sensitivity of 0.77 (95% CI 0.65‐0.89), specificity of 0.60 (95% CI 0.58‐0.62), AUC of 0.73 (95% CI 0.66‐0.79), and MCC of 0.11 (95% CI 0.07‐0.14), near-perfect performance on resampled training data (AUC 0.98) indicated pronounced overfitting. This effect was further amplified in the incremental XGBoost implementation, which showed slightly reduced test performance (sensitivity 0.70, specificity 0.62; AUC 0.68; MCC 0.09) alongside increased overfitting.

##### SVM Model

SVM achieved the highest overall sensitivity of 0.79 (95% CI 0.67‐0.90). However, compared with the SGD model, this was accompanied by lower specificity (0.60; 95% CI 0.58‐0.62) and AUC (0.74; 95% CI 0.67‐0.80). Compared with boosting- and tree-based approaches, SVM exhibited less overfitting, maintaining stable discriminatory performance under the 2% incidence setting. Notably, we observed relatively stable SVM performance across all resampling methods.

Figure S3 in Multimedia Appendix 2 illustrates the performance of models across decision thresholds between 0.01 and 0.05. As expected, increasing the decision threshold resulted in a decrease in sensitivity and a corresponding increase in specificity. LR, SGD, and boosting models exhibited stable and monotonic sensitivity-specificity trade-offs, with SGD demonstrating an optimal balance between 0.02 and 0.03.

### Data Distribution Analysis

**Figure 4.**
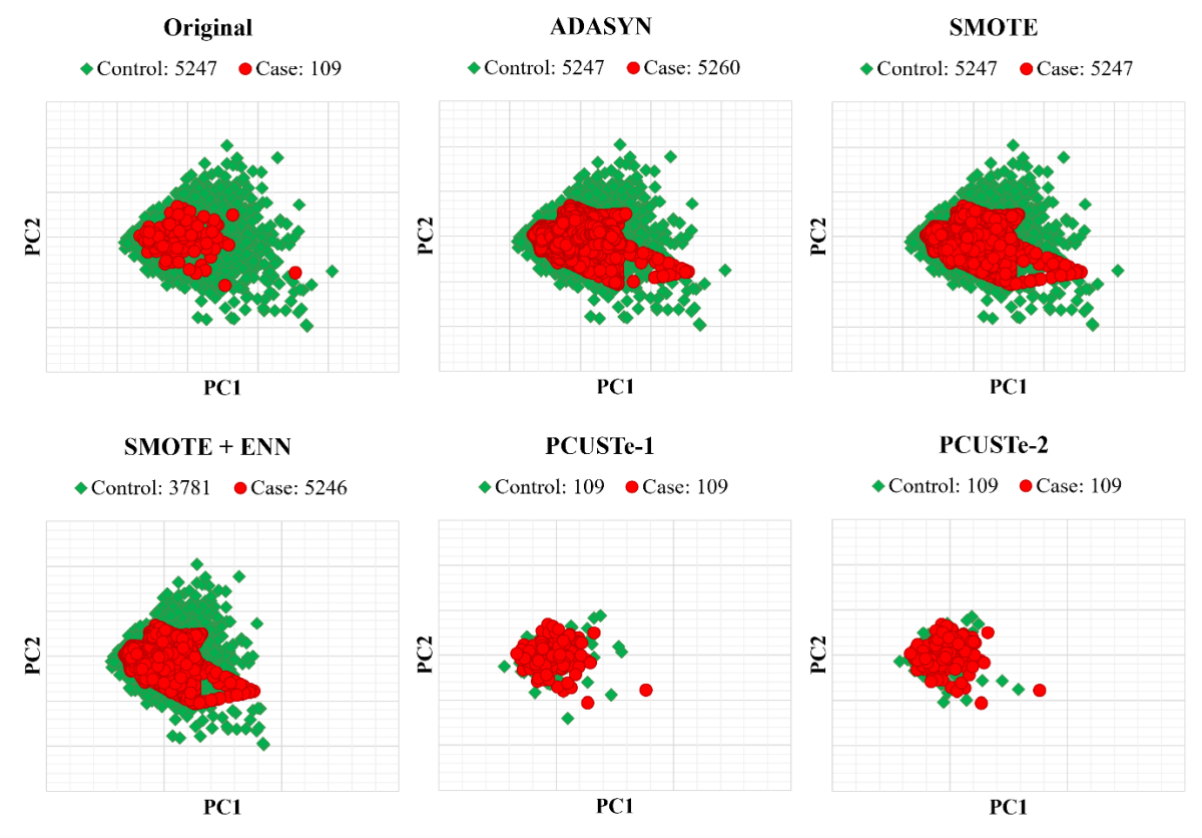
Principal component analysis visualization of dataset distributions across various resampling strategies. To ensure a consistent baseline, the principal component analysis transformation was fitted on the original training data and applied across all subsets. Top row: original imbalanced distribution and popular oversampling methods. Bottom row: hybrid resampling via SMOTE+ edited nearest neighbors, and the proposed PCUSTe methods. Red markers represent gastrointestinal cancer cases, and green markers represent controls. Case and control counts for each method are indicated in the respective subplot legends. ADASYN: adaptive synthetic sampling; PCUSTe-1: PCUSTe with sociodemographic matching (education, employment, income, and marital status); PCUSTe-2: PCUSTe with lifestyle matching (smoking and drinking); SMOTE: synthetic minority oversampling.

The PCA projections provide a top-level visual comparison of how each resampling method altered the class proportions within the primary components of variance (Figure 4). In the original dataset, the minority class is concentrated within a specific region already occupied by a high density of controls. The oversampling techniques increased the visibility of the minority class by increasing point density within those existing regions. In contrast, the undersampling methods did not alter the minority class but reduced the majority class instead. Notably, SMOTE+ ENN aggressively reduced the control population—resulting in a dataset that was no longer balanced (with cases becoming the majority). Across all methods, the 2D projections suggest that resampling primarily shifts the relative density of the classes rather than creating clear linear separation in the first 2 principal components.

The distance heatmap evaluates the mean absolute predictor-wise difference between cases and controls (Figure S4 in Multimedia Appendix 2). Both PCUSTe-1 and PCUSTe-2 markedly reduced distances for age and several additional biological variables, despite these factors not being explicitly used for proportional matching. Distances between fatty acids and some micronutrients were increased instead. This suggests that matching based on socioeconomic variables (PCUSTe-1) or lifestyle factors (PCUSTe-2) indirectly aligns unselected physiological characteristics between cases and controls. In contrast, SMOTE+ ENN increased overall distances for age, blood pressure, and multiple other predictors.

**Figure 5.**
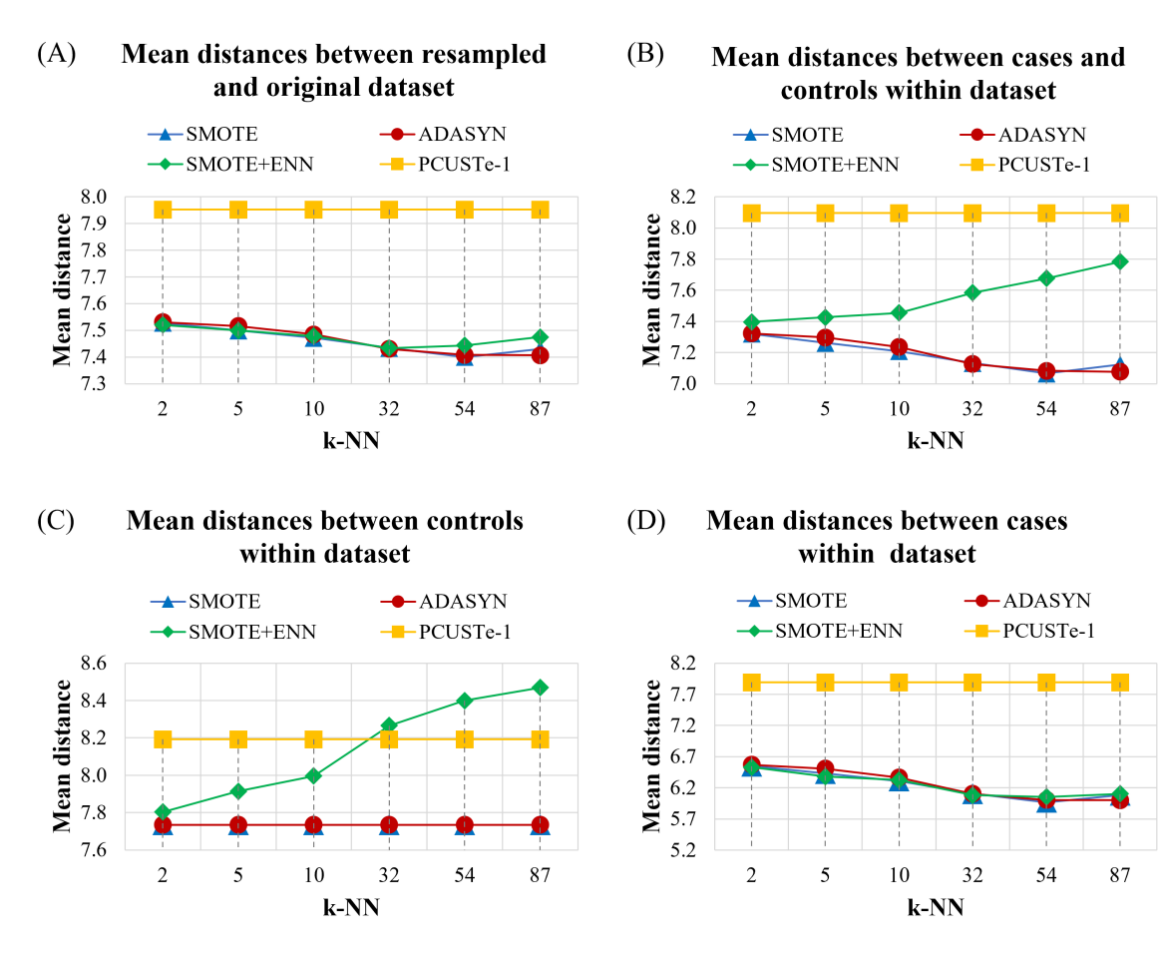
Comparison of mean distances among different k-NN in oversampled datasets using SMOTE, ADASYN, and SMOTE+ ENN. PCUSTe-1 is included for comparison. (A) Mean distances between resampled and original datasets across k values (top-left); (B) Mean distances between cases and controls within each resampled dataset (top-right); (C) Mean distances between control samples in each resampled dataset (bottom-left); (D) Mean distances between case samples in resampled datasets (bottom-right). ADASYN: adaptive synthetic sampling; ENN: edited nearest neighbors; k-NN: k-nearest neighbors; PCUSTe-1: PCUSTe with sociodemographic matching (education, employment, income, and marital status); SMOTE: synthetic minority oversampling.

The comparative analysis across varying k-NN values demonstrated that SMOTE and ADASYN exhibit highly similar behavior, generally maintaining the lowest mean distances to the original dataset and the narrowest gaps between classes (Figure 5). In contrast, SMOTE+ ENN showed a distinct upward trend in both case-control and control-control distances as the k-NN parameter increases. This suggests that the ENN cleaning process becomes more aggressive at higher neighbor scales, significantly increasing data sparsity and class separability by removing ambiguous majority instances. The PCUSTe-1 undersampling method remained constant as it is k-independent. Notably, PCUSTe-1 maintained significantly larger mean distances for cases compared with oversampling methods—this is expected, as oversampling generates synthetic samples in close proximity to original minority observations—whereas undersampling preserves the sparse, natural distribution of the original cases.

## Discussion

### Principal Findings

Our analysis highlights that extreme class imbalance materially affects ML performance in cohort-based GI cancer prediction. In our experiments, extensive oversampling—even after k-NN parameter tuning—resulted in overfitting in complex nonlinear classifiers (RF, XGBoost, and SVM), whereas linear classifiers (LR and SGD) were comparatively robust. We developed an undersampling method, PCUSTe, which achieved stable test set performance across multiple classifiers without relying on synthetic data. To mitigate the reduction in training data inherent to undersampling, we combined PCUSTe with an incrementally trained SGD model. Among all evaluated configurations, this approach yielded the greatest improvement in predictive performance. The model with the highest performance achieved an AUC of 0.77 (95% CI 0.70‐0.84) and sensitivity of 0.77 (95% CI 0.64‐0.89), while maintaining moderate specificity (0.65, 95% CI 0.63‐0.67). Importantly, our findings indicate that the application of resampling techniques to address extreme class imbalance should be tailored to specific study objectives. In this study, we prioritized sensitivity over specificity, consistent with the objective of identifying individuals at elevated GI cancer risk who may benefit from modification of dietary and early metabolic risk factors. Conversely, in settings where a high false-positive rate may impose a substantial burden on participants, threshold adjustment alone may be preferable to resampling-based approaches.

### Comparison to Previous Work

Cohort studies provide longitudinal insights into individual health trajectories prior to disease onset, offering valuable data for identifying early risk factors and informing preventive strategies. However, for diseases that are uncommon in the general population, class imbalance remains a persistent challenge. A US-based study using blood count data to predict GI cancers achieved moderate discrimination (area under the receiver operating characteristic curve [AUROC]=0.75) but a low *F*_1_-score (0.03) when addressing imbalance solely via class weights [47]. Similarly, although models such as XGBoost and SVM can outperform linear classifiers when combined with class weighting, they are not inherently robust to extreme imbalance [48,49].

In our study, nonlinear models trained without resampling yielded low to moderate sensitivity, often improving sensitivity at the expense of specificity or vice versa. For example, the XGBoost model trained on nonresampled data achieved only 0.06 sensitivity and 0.98 specificity, whereas the SVM trained on the same data showed 0.64 sensitivity and 0.54 specificity. Performance for both algorithms improved substantially when models were trained on data resampled using the PCUSTe-2 method. For instance, the XGBoost model trained on this dataset achieved a sensitivity of 0.66 with a specificity of 0.61 prior to tuning and sensitivity of 0.73 with specificity of 0.63 after tuning. These findings suggest that, in settings of extreme imbalance, algorithm choice alone may be insufficient to achieve adequate minority-class detection.

Resampling has therefore emerged as one of the most commonly used strategies to mitigate class imbalance. For instance, oversampling is frequently applied in biomedical ML model training, and previous studies have reported improved cancer prediction using methods such as SMOTE and its variants [50,51]. Although oversampling can help address moderate imbalance, the aggressive oversampling required to correct severe imbalance remains challenging, particularly when complex ML algorithms are used. For example, a Korean study using national health insurance data to predict gastric cancer in adults (65,657 cases and 10,450,292 controls) used the Random Oversampling Examples (ROSE) method to address imbalance [52]. The authors reported the best performance with an LR model (AUROC 0.71) compared with decision tree and XGBoost classifiers. This aligns with our findings, where LR models trained on oversampled data consistently outperformed more complex classifiers trained on the same data.

When comparing performance across resampling strategies, complex models showed clear signs of overfitting, particularly on oversampled data. Overfitting in such datasets is well documented and remains an ongoing challenge despite numerous mitigation attempts [53]. During oversampling with the default k-NN setting (k=5), synthetic minority instances are generated in very close proximity to the original samples. As shown in our data distribution analyses, the resulting datasets did not create a clear linear separation in the first 2 principal components but instead shifted the relative density of the classes. Larger k values produced sparser training datasets and reduced overfitting. Although oversampling methods are widely used in biomedical ML, many studies rely on default parameter settings. Our findings extend prior work by demonstrating that k-NN tuning substantially influences model performance, with higher k values potentially yielding more stable and accurate predictions.

In contrast, models trained on undersampled data may be less prone to overfitting but are limited to a reduced training size. By combining undersampling with an incremental SGD model, we attempted to address this limitation and expose models to a larger proportion of the original training dataset. Our results show that this approach improved sensitivity while maintaining moderate specificity. Compared with other evaluated models trained on undersampled data, this combined strategy produced better performance. However, due to the customized implementation, model tuning was difficult, and our tuning strategy did not improve test performance relative to the crude model, suggesting that more sophisticated tuning approaches may be required to further improve baseline performance.

When comparing performance across ML algorithms, our results showed a clear distinction between linear and more complex classifiers in terms of overfitting behavior. Prior studies have shown that RFs and boosted trees are particularly susceptible to overfitting in small or noisy datasets. For instance, a recent simulation study demonstrated that deep trees can form local probability “peaks” around training samples, inflating apparent discrimination while reducing generalizability [54]. Interestingly, that study also found that RF models, despite overfitting the training data, could still achieve strong test performance. Consistent with these findings, although high training accuracy suggested overfitting, RF models trained on undersampled data maintained strong test-set performance in our study. When trained on oversampled data, RF models likely formed localized decision regions around dense clusters of synthetic minority samples, which may have led to much of the remaining feature space being classified as the majority class. Performance improved when a larger k parameter and ENN cleaning were applied, likely because increased inter-sample distances produced a more dispersed minority distribution and reduced this form of overfitting. In contrast, the SGD model exhibited benign underfitting, a form of regularization that limits training accuracy but enhances generalization [55].

Several studies have raised concerns about data resampling approaches due to their potential to distort population structure and adversely affect model calibration [56]. Decision threshold adjustment, therefore, emerges as an alternative strategy for addressing class imbalance. However, selecting an appropriate decision threshold remains challenging, and a variety of automated threshold-search methods have been proposed [57]. Because this study focuses on strategies to mitigate class imbalance in GI cancer binary classification, we adopted the observed disease incidence as the decision threshold. Although the exact incidence in a target population cannot be known with certainty, estimates from epidemiologic studies are widely available and may be more appropriate for risk stratification in biomedical settings than thresholds derived from purely algorithmic optimization.

Despite achieving strong performance with decision threshold adjustment alone for selected models, PCUSTe-based training set resampling improved minority-class detection across most configurations, particularly among more complex learning algorithms. Both evaluated configurations—PCUSTe-1 (matching on marital status, education, employment, and income) and PCUSTe-2 (matching on smoking and alcohol consumption)—supported favorable predictive performance, consistent with established associations between socioeconomic, lifestyle factors, and cancer risk [58,59]. Importantly, PCUSTe’s flexible parameterization—defined by its matching criteria and case-control ratio—allows adaptation to different datasets and research contexts. We observed that linear models tended to perform better under PCUSTe-1 resampling, whereas more complex classifiers showed improved performance with PCUSTe-2. When considered alongside SHAP analyses, which indicated stronger contributions of dietary factors in linear models and greater influence of physiological signals in nonlinear models, this pattern may suggest that the 2 matching strategies emphasize distinct aspects of GI cancer risk. Collectively, these findings reinforce that no single ML algorithm or imbalance mitigation strategy is universally optimal. Instead, adaptable modeling frameworks capable of efficient retraining across populations may be better suited to support precision prevention in nutrition and cancer research [60].

In ML, large sample sizes are often favored, and some studies address data sparsity by pooling multiple data sources. For example, a recent Danish study combined 3 national registries and full-text medical records to develop a multi-cancer prediction model comprising 6.7 million individuals. Although internal validation showed strong performance for GI cancers (AUCs: pancreatic 0.86, liver 0.90, colorectal 0.85, gastric 0.85, oesophageal 0.89), external validation using the UK Biobank yielded substantially lower AUCs (0.65‐0.74) [61]. Supported by multiple large-scale grants, this study illustrates the substantial resources required for population-level modeling. In contrast, our study used a smaller, grouped GI cancer dataset and has not yet undergone external validation. While direct comparison is limited due to differences in sample size and feature composition, our models nonetheless demonstrated promising predictive performance. These findings suggest that careful resampling and study design may partially mitigate limitations inherent to small, highly imbalanced datasets, although they cannot substitute for large-scale population data.

### Strengths and Limitations

This study has several notable strengths. First, we leveraged longitudinal cohort data, enabling a robust assessment of risk factors preceding disease onset, and implemented a rigorous evaluation framework with strict separation between training and test datasets. Second, we introduced PCUSTe, a patient-centered undersampling framework that flexibly parameterizes matching criteria, offering a customizable, epidemiologically grounded approach to addressing class imbalance in small biomedical datasets. Third, we conducted a comprehensive comparison of resampling strategies across multiple ML algorithms and hyperparameter configurations.

Several limitations should also be acknowledged. First, dietary intake data were self-reported, introducing potential recall bias, and the lack of repeated dietary assessments limited our ability to capture temporal changes in exposure. To partially mitigate this, analyses were restricted to baseline assessments collected prior to diagnosis using standardized instruments. Second, although internal validation was performed, external validation is required to assess generalizability. While this limitation was partially addressed through conservative model evaluation, validation in independent cohorts remains an important next step. Third, the relatively small number of GI cancer cases may have constrained model performance and limited extrapolation to broader populations. We attempted to mitigate this by using multiple resampling strategies and applying class-prior-based decision threshold adjustment; however, larger sample sizes are needed to confirm the stability of the observed results.

In addition, the limited number of positive cases precluded robust modeling of site-specific GI cancer subtypes, necessitating a binarized pooled outcome. Although the final models did not appear to systematically misclassify risk for any specific GI cancer site, the biological and clinical heterogeneity of these malignancies should be acknowledged. Future analyses incorporating multi-site classification will be needed once sufficient sample sizes become available. Finally, while PCUSTe provides a promising solution for smaller cohorts, it further reduces the control population, and models trained on limited samples are inherently less competitive than those developed using large-scale datasets. To mitigate this, we paired PCUSTe with an incrementally trained SGD model, allowing iterative learning from multiple control subsets constrained by case covariate distributions. Nevertheless, caution is warranted when extrapolating model performance to broader clinical settings.

### Future Work

While PCUSTe and incidence-based threshold adjustment offer a practical and adaptable framework for improving risk stratification in cohort-based cancer prediction studies, external validation in larger and more diverse cohorts is required to establish the generalizability and scalability of these methods.

### Conclusions

This study demonstrates that a class imbalance mitigation strategy is a critical determinant of ML model performance in GI cancer risk prediction. By incorporating epidemiologically grounded matching principles, our patient-centered undersampling framework, PCUSTe, consistently outperformed conventional oversampling and hybrid approaches across multiple model architectures. However, incidence-based decision threshold adjustment alone, paired with a baseline LR model, showed better balance between sensitivity and specificity metrics. These findings underscore the value of aligning ML workflows with real-world population characteristics to enhance minority class detection in small, highly imbalanced datasets typical of rare or low-incidence disease research. Furthermore, the choice of the best class imbalance mitigation strategy may not be uniform across different study objectives.

## Supplementary material

10.2196/78931 Multimedia Appendix 1 Patient Centered Undersampling technique (PCUSTe) pseudocode.

10.2196/78931 Multimedia Appendix 2 Supplementary tables and figures.

## Abbreviations

ADASYN: adaptive synthetic sampling
AUC: area under the receiver operating characteristic curve
AUROC: area under the receiver operating characteristic curve
DBP: diastolic blood pressure
ENN: edited nearest neighbors
EPV: event per variable
GI: gastrointestinal
*ICD-10*: *International Statistical Classification of Diseases, Tenth Revision*
k-NN: k-nearest neighbor
KNCC: Korean National Cancer Center
LR: logistic regression
MCC: Matthews correlation coefficient
ML: machine learning
PCA: principal component analysis
PCUSTe: patient-centered undersampling technique
PCUSTe-1: PCUSTe with sociodemographic matching (education, employment, income, and marital status)
PCUSTe-2: PCUSTe with lifestyle matching (smoking and drinking)
RF: random forest
ROSE: Random Oversampling Examples
RS: RandomizedSearchCV
SBP: systolic blood pressure
SGD: stochastic gradient descent
SHAP: Shapley additive explanations
SMOTE: synthetic minority over-sampling technique
SVM: support vector machine
XGBoost: extreme gradient boosting

## References

[1] Arnold M, Abnet CC, Neale RE (2020). Global burden of 5 major types of gastrointestinal cancer. Gastroenterology.

[2] Duan B, Zhao Y, Bai J, Morgado-Diaz JA (2022). Gastrointestinal Cancers.

[3] Thuler LCS, Morgado-Diaz JA (2022). Gastrointestinal Cancers.

[4] Bray F, Laversanne M, Sung H (2024). Global cancer statistics 2022: GLOBOCAN estimates of incidence and mortality worldwide for 36 cancers in 185 countries. CA Cancer J Clin.

[5] Shin WS, Xie F, Chen B (2023). Updated epidemiology of gastric cancer in Asia: decreased incidence but still a big challenge. Cancers (Basel).

[6] Kratz JD, Klein AB, Gray CB (2024). The epidemiology of biliary tract cancer and associated prevalence of MDM2 amplification: a targeted literature review. Target Oncol.

[7] Jiang D, Wu Y, Liu L (2024). Burden of gastrointestinal tumors in Asian countries, 1990-2021: an analysis for the global burden of disease study 2021. Clin Epidemiol.

[8] Morgan E, Arnold M, Camargo MC (2022). The current and future incidence and mortality of gastric cancer in 185 countries, 2020-40: a population-based modelling study. EClinicalMedicine.

[9] Moons KGM, Royston P, Vergouwe Y, Grobbee DE, Altman DG (2009). Prognosis and prognostic research: what, why, and how?. BMJ.

[10] Altman DG (2009). Prognostic models: a methodological framework and review of models for breast cancer. Cancer Invest.

[11] Moons KGM, Wolff RF, Riley RD (2019). PROBAST: a tool to assess risk of bias and applicability of prediction model studies: explanation and elaboration. Ann Intern Med.

[12] Zhang J, Chen Q, Zhang Y, Zhou J (2024). Construction of a random survival forest model based on a machine learning algorithm to predict early recurrence after hepatectomy for adult hepatocellular carcinoma. BMC Cancer.

[13] Tran TT, Lee J, Gunathilake M (2023). A comparison of machine learning models and Cox proportional hazards models regarding their ability to predict the risk of gastrointestinal cancer based on metabolic syndrome and its components. Front Oncol.

[14] Kim H, Park T, Jang J, Lee S (2022). Comparison of survival prediction models for pancreatic cancer: cox model versus machine learning models. Genomics Inform.

[15] Kang SU, Nam SJ, Kwon OB (2024). Predictive mortality and gastric cancer risk using clinical and socio-economic data: a nationwide multicenter cohort study. Cancers (Basel).

[16] Miglietta F, Collesei A, Vernieri C (2025). Development of two machine learning models to predict conversion from primary HER2-0 breast cancer to HER2-low metastases: a proof-of-concept study. ESMO Open.

[17] Menardi G, Torelli N (2014). Training and assessing classification rules with imbalanced data. Data Min Knowl Disc.

[18] Rahman A, Nahid N, Schuller B, Ahad MAR (2024). A stacked CNN and random forest ensemble architecture for complex nursing activity recognition and nurse identification. Sci Rep.

[19] Triantafillidis JK, Georgiou K, Konstadoulakis MM, Papalois AE (2024). Early-onset gastrointestinal cancer: An epidemiological reality with great significance and implications. World J Gastrointest Oncol.

[20] Poorolajal J, Moradi L, Mohammadi Y, Cheraghi Z, Gohari-Ensaf F (2020). Risk factors for stomach cancer: a systematic review and meta-analysis. Epidemiol Health.

[21] Ilic I, Zivanovic Macuzic I, Ravic-Nikolic A, Ilic M, Milicic V (2024). Global burden of esophageal cancer and its risk factors: a systematic analysis of the global burden of disease study 2019. Life.

[22] Roshandel G, Ghasemi-Kebria F, Malekzadeh R (2024). Colorectal cancer: epidemiology, risk factors, and prevention. Cancers (Basel).

[23] Hu JX, Zhao CF, Chen WB (2021). Pancreatic cancer: a review of epidemiology, trend, and risk factors. World J Gastroenterol.

[24] Li Y, Ou Z, Yu D (2023). The trends in death of primary liver cancer caused by specific etiologies worldwide: results from the global burden of disease study 2019 and implications for liver cancer management. BMC Cancer.

[25] Moglia V, Johnson O, Cook G, de Kamps M, Smith L (2025). Artificial intelligence methods applied to longitudinal data from electronic health records for prediction of cancer: a scoping review. BMC Med Res Methodol.

[26] Chawla NV, Bowyer KW, Hall LO, Kegelmeyer WP (2002). SMOTE: synthetic minority over-sampling technique. JAIR.

[27] Kim J (2014). Cancer screenee cohort study of the national cancer center in South Korea. Epidemiol Health.

[28] Breu AC, Patwardhan VR, Nayor J (2019). A multicenter study into causes of severe acute liver injury. Clin Gastroenterol Hepatol.

[29] Xing M, Gao M, Li J, Han P, Mei L, Zhao L (2022). Characteristics of peripheral blood Gamma-glutamyl transferase in different liver diseases. Medicine (Baltimore).

[30] Parhofer KG, Laufs U (2019). The diagnosis and treatment of hypertriglyceridemia. Dtsch Arztebl Int.

[31] Jung M, Ha E, Kwon O, Kim H (2023). Development of a semi-quantitative food frequency questionnaire for dietary intake of elementary school children: data from the Seventh Korea national health and nutrition examination survey. Nutr Res Pract.

[32] Yun SH, Shim JS, Kweon S, Oh K (2013). Development of a food frequency questionnaire for the Korea National health and nutrition examination survey: data from the fourth Korea national health and nutrition examination survey (KNHANES IV). Korean J Nutr.

[33] Willett WC, Howe GR, Kushi LH (1997). Adjustment for total energy intake in epidemiologic studies. Am J Clin Nutr.

[34] Friedewald WT, Levy RI, Fredrickson DS (1972). Estimation of the concentration of low-density lipoprotein cholesterol in plasma, without use of the preparative ultracentrifuge. Clin Chem.

[35] Jankovic S (2022). Tests for comparison of two groups: student’s T-test, Mann-Whitney U-test and chi-square test. Int J Biomed Healthc.

[36] Vittinghoff E, McCulloch CE (2007). Relaxing the rule of ten events per variable in logistic and Cox regression. Am J Epidemiol.

[37] Peng CYJ, Lee KL, Ingersoll GM (2002). An introduction to logistic regression analysis and reporting. J Educ Res.

[38] Bottou L, Montavon G, Orr GB, Müller KR (2012). Neural Networks: Tricks of the Trade.

[39] Breiman L (2001). Random forests. Mach Learn.

[40] Chen T, Guestrin C XGBoost: a scalable tree boosting system.

[41] Hearst MA, Dumais ST, Osuna E, Platt J, Scholkopf B (1998). Support vector machines. IEEE Intell Syst Their Appl.

[42] Lundberg SM, Lee SI A unified approach to interpreting model predictions. https://proceedings.neurips.cc/paper/2017/file/8a20a8621978632d76c43dfd28b67767-Paper.pdf.

[43] Chicco D, Jurman G (2020). The advantages of the Matthews correlation coefficient (MCC) over F1 score and accuracy in binary classification evaluation. BMC Genomics.

[44] Bekkar M, Djemaa HK, Alitouche TA (2013). Evaluation measures for models assessment over imbalanced data sets. J Inf Eng Appl.

[45] Pozzolo AD, Caelen O, Johnson RA, Bontempi G Calibrating probability with undersampling for unbalanced classification.

[46] Everitt B (2011). Introduction to Applied Multivariate Analysis with R.

[47] Read AJ, Zhou W, Saini SD, Zhu J, Waljee AK (2023). Prediction of gastrointestinal tract cancers using longitudinal electronic health record data. Cancers (Basel).

[48] Jung KM (2015). Support vector machines for unbalanced multicategory classification. Math Probl Eng.

[49] Yan Z, Wen H (2021). Electricity theft detection base on extreme gradient boosting in AMI. IEEE Trans Instrum Meas.

[50] Muraru MM, Simó Z, Iantovics LB (2024). Cervical cancer prediction based on imbalanced data using machine learning algorithms with a variety of sampling methods. Appl Sci (Basel).

[51] Alsmariy R, Healy G, Abdelhafez H (2020). Predicting cervical cancer using machine learning methods. IJACSA.

[52] Park B, Kim CH, Jun JK (2025). A machine learning risk prediction model for gastric cancer with Shapley additive explanations. Cancer Res Treat.

[53] Alkhawaldeh IM, Albalkhi I, Naswhan AJ (2023). Challenges and limitations of synthetic minority oversampling techniques in machine learning. World J Methodol.

[54] Barreñada L, Dhiman P, Timmerman D, Boulesteix AL, Van Calster B (2024). Understanding overfitting in random forest for probability estimation: a visualization and simulation study. Diagn Progn Res.

[55] Koren T, Livni R, Mansour Y, Sherman U (2022). Benign underfitting of stochastic gradient descent. http://www.proceedings.com/68431.html.

[56] van den Goorbergh R, van Smeden M, Timmerman D, Van Calster B (2022). The harm of class imbalance corrections for risk prediction models: illustration and simulation using logistic regression. J Am Med Inform Assoc.

[57] Esposito C, Landrum GA, Schneider N, Stiefl N, Riniker S (2021). GHOST: adjusting the decision threshold to handle imbalanced data in machine learning. J Chem Inf Model.

[58] Biesbroek S, Kneepkens MC, van den Berg SW (2018). Dietary patterns within educational groups and their association with CHD and stroke in the European prospective investigation into cancer and nutrition-Netherlands cohort. Br J Nutr.

[59] da Costa GG, da Conceição Nepomuceno G, da Silva Pereira A, Simões BFT (2022). Worldwide dietary patterns and their association with socioeconomic data: an ecological exploratory study. Global Health.

[60] Stultz CM (2023). Machine learning for risk prediction: does one size really fit all?. JACC Adv.

[61] Jung AW, Holm PC, Gaurav K (2024). Multi-cancer risk stratification based on national health data: a retrospective modelling and validation study. Lancet Digit Health.

