# Supplementary material for: Advancing Gastrointestinal Cancer Risk Prediction With Patient-Centered Machine Learning: Machine Learning Modeling Study
Source: JMIR Med Inform. 2026 Jun 4;14:e78931. doi: 10.2196/78931 (PMC13235658; doi:10.2196/78931)
Supplement: Multimedia Appendix 1 [file medinform-v14-e78931-s001.pdf]

Multimedia Appendix 1

PCUSTe – Patient Centered Under-sampling technique

Input:

- Dataset $\mathcal{D} = \{(x_i, y_i)\}_{i=1}^{N}$
- Matching feature set $\mathcal{M} = \{M_1, M_2, \dots, M_k\}$
- Binary target variable $y_i \in \{0,1\}$
- Control-to-case ratio r
- Random seed ξ

Output:

A balanced dataset $\mathcal{D}_{\text{PCUSTe}}$ consisting of all case samples and proportionally selected control samples.

Procedure:

1. Partition dataset.
   Separate cases and controls:

$$\mathcal{C} = \{x_i \in \mathcal{D}: y_i = 1\}, \mathcal{N} = \{x_i \in \mathcal{D}: y_i = 0\}$$

   Let $n_c = | \mathcal{C} |$and $n_n = | \mathcal{N} |$.

2. Define target control count.

$$n_n^* = r \cdot n_c$$

3. Group cases by matching features.
   Use $\mathcal{M}$ to form groups

$$\mathcal{G} = \{g_1, g_2, \dots, g_G\}$$

   where each g_j represents a unique combination of categorical values in $\mathcal{M}$.

4. Compute case proportions per group.
   For each $g_j \in \mathcal{G}$:

$$p_j = \frac{| \{x_i \in \mathcal{C}: g(x_i) = g_j\} |}{n_c}$$

5. Initialize empty sets.
   - $\widetilde{\mathcal{N}} = \emptyset$ (selected controls)
   - $U = \emptyset$ (used control indices)

6. Iterate over each group g_j.
   (a) Identify available controls in the same matching stratum:
$$\mathcal{N}_{g_j} = \{x_i \in \mathcal{N}: g(x_i) = g_j\} \setminus U$$
   (b) Determine number of controls to draw:
$$n_j = \min(\lfloor p_j \cdot n_n^* \rceil, | \mathcal{N}_{g_j} |)$$
   (c) If $| \mathcal{N}_{g_j} | > 0$, then randomly sample without replacement:
$$\widetilde{\mathcal{N}}_{g_j} = \text{SampleWithoutReplacement}(\mathcal{N}_{g_j}, n_j; \xi)$$
   (d) Add sampled controls to selected set:
$$\widetilde{\mathcal{N}} \leftarrow \widetilde{\mathcal{N}} \cup \widetilde{\mathcal{N}}_{g_j}$$
   (e) Mark their indices as used:
$$U \leftarrow U \cup \widetilde{\mathcal{N}}_{g_j}$$
7. Combine cases and selected controls.
$$\mathcal{D}^* = \mathcal{C} \cup \widetilde{\mathcal{N}}$$
8. Shuffle dataset.
   Randomly permute all samples using seed ξ:
$$\mathcal{D}_{\text{PCUSTe}} = \text{Shuffle}(\mathcal{D}^*; \xi)$$
9. Return balanced dataset.
$$\text{Return } \mathcal{D}_{\text{PCUSTe}}$$

Notes:

- The algorithm ensures that the proportional distribution of case strata (based on $\mathcal{M}$) is maintained in the sampled controls.
- Each control sample can appear only once (no replacement).
- Random seed ξ ensures reproducibility across runs.
- The output dataset has an overall control-to-case ratio of approximately $r: 1$, but proportions are matched by stratum.

Notation Definition:

$$\text{SampleWithoutReplacement}(A, n; \xi)$$

denotes uniform random selection of n distinct elements from set A without replacement, using random seed ξ.
