# Supplementary material for: Advancing Gastrointestinal Cancer Risk Prediction With Patient-Centered Machine Learning: Machine Learning Modeling Study
Source: JMIR Med Inform. 2026 Jun 4;14:e78931. doi: 10.2196/78931 (PMC13235658; doi:10.2196/78931)
Supplement: Multimedia Appendix 2 [file medinform-v14-e78931-s002.pdf]

Multimedia Appendix 2

Table S1. Descriptive statistics of test and train datasets - categorical variables. [a]

Characteristics	Test data			Train data		
	Case (n=47)	Control (n=2249)	*P*	Case (n=109)	Control (n=5247)	*P*
Gender, n (%)			<.001			<.001
Male	29 (61.7)	776 (34.5)		60 (55.0)	1914 (36.5)	
Female	18 (38.3)	1473 (65.5)		49 (45.0)	3333 (63.5)	
Alcohol consumer, n (%)			.97			.53
No	18 (38.3)	830 (36.9)		37 (33.9)	1960 (37.4)	
Yes	29 (61.7)	1419 (63.1)		72 (66.1)	3287 (62.6)	
Smoker, n (%)			.002			.005
No	21 (44.7)	1511 (67.2)		58 (53.2)	3492 (66.6)	
Yes	26 (55.3)	738 (32.8)		51 (46.8)	1755 (33.4)	
Married or cohabitating, n (%)			.30			.44
No	3 (6.4)	281 (12.5)		16 (14.7)	620 (11.8)	
Yes	44 (93.6)	1968 (87.5)		93 (85.3)	4627 (88.2)	
Unemployed			.86			.57
No	26 (55.3)	1298 (57.7)		66 (60.6)	3009 (57.3)	
Yes	21 (44.7)	951 (42.3)		43 (39.4)	2238 (42.7)	
Higher education [b], n (%)			.75			.20
No	27 (57.4)	1214 (54.0)		65 (59.6)	2780 (53.0)	
Yes	20 (42.6)	1035 (46.0)		44 (40.4)	2467 (47.0)	
Lower income [c], n (%)			.11			.006
No	33 (70.2)	1815 (80.7)		75 (68.8)	4194 (79.9)	
Yes	14 (29.8)	434 (19.3)		34 (31.2)	1053 (20.1)	

[a] Values are reported as frequencies and proportion. *P*< .05 was considered as statistically significant.
[b] Higher education: college or above.
[c] Lower income: below 2 million Korean won per month.

Table S2. Descriptive statistics of test and train datasets - continuous variables. [a]

Characteristic	Test data			Train data		
	Case (n=47)	Control (n=2249)	*P*	Case (n=109)	Control (n=5247)	*P*
Age (years), mean (SD)	57.43 (7.61)	52.19 (8.23)	<.001	57.55 (7.96)	52.40 (8.19)	<.001
AST [b] (U/L), mean (SD)	28.85 (16.89)	22.81 (11.16)	.02	28.47 (14.29)	23.04 (11.10)	<.001
BMI [c] (kg/m^2), mean (SD)	24.75 (2.49)	23.52 (3.00)	.002	25.06 (3.18)	23.62 (2.96)	<.001
Calcium (mg/day), mean (SD)	486.35 (208.43)	485.54 (209.16)	.98	469.72 (196.98)	487.46 (212.84)	.36
Carbohydrate (g/day), mean (SD)	309.33 (37.24)	314.17 (34.12)	.38	318.14 (32.19)	314.05 (33.54)	.19
Carotene (μg/day), mean (SD)	1912.19 (775.27)	2103.78 (1193.93)	.10	2004.81 (1116.94)	2137.01 (1233.79)	.23
Cholesterol (mg/day), mean (SD)	140.98 (99.35)	131.96 (78.05)	.54	112.52 (59.41)	131.59 (76.67)	.001
DBP [d] (mmHg), mean (SD)	81.21 (10.28)	75.77 (10.52)	<.001	82.06 (11.08)	76.14 (10.46)	<.001
Fasting blood glucose (mg/dL), mean (SD)	109.96 (52.40)	92.88 (14.15)	.03	99.19 (20.51)	93.04 (14.19)	.002
Fat (g/day), mean (SD)	31.28 (13.31)	30.50 (11.75)	.70	28.28 (11.00)	30.45 (11.43)	.04
Fiber (g/day), mean (SD)	15.06 (7.46)	16.69 (7.60)	.14	15.16 (6.25)	16.69 (7.42)	.01
GGT [e] (IU/L), mean (SD)	45.26 (43.71)	27.98 (29.95)	.01	44.13 (76.75)	29.14 (35.24)	.04
HDL [f] (mg/dL), mean (SD)	54.43 (11.78)	59.75 (14.53)	.004	55.97 (13.53)	59.76 (14.61)	.005
Iron (mg/day), mean (SD)	10.32 (2.87)	10.31 (2.83)	.99	10.34 (2.48)	10.36 (2.90)	.95
LDL [g] (mg/dL), mean (SD)	110.49 (39.54)	117.01 (32.55)	.27	115.73 (34.63)	116.93 (32.66)	.72
Magnesium (mg/day), mean (SD)	163.49 (64.15)	177.09 (67.99)	.16	170.11 (66.93)	177.57 (67.85)	.25
MUFA [h] (g/day), mean (SD)	8.22 (4.62)	8.10 (4.08)	.85	7.15 (3.26)	8.01 (3.86)	.008
Niacin (mg/day), mean (SD)	9.75 (2.24)	10.27 (2.55)	.12	9.83 (2.50)	10.33 (2.61)	.04
Phosphorus (mg/day), mean (SD)	892.74 (214.15)	896.74 (219.27)	.90	867.33 (202.97)	901.00 (223.10)	.09
Potassium (mg/day), mean (SD)	2303.40 (651.90)	2452.76 (793.85)	.13	2326.52 (759.88)	2463.96 (802.85)	.07
Protein (g/day), mean (SD)	63.37 (11.68)	62.33 (11.23)	.55	61.24 (10.28)	62.56 (11.49)	.19
PUFA [i] (g/day), mean (SD)	4.60 (2.11)	4.86 (2.09)	.42	4.60 (2.01)	4.83 (2.05)	.23
SBP [j] (mmHg), mean (SD)	131.21 (12.33)	124.40 (14.60)	<.001	131.97 (16.15)	124.86 (14.40)	<.001
SFA [k] (g/day), mean (SD)	9.39 (4.60)	9.05 (4.36)	.62	8.04 (3.72)	9.03 (4.24)	.007
Sodium (mg/day), mean (SD)	2010.63 (846.99)	1967.86 (758.34)	.73	1982.49 (804.22)	1987.98 (787.04)	.94

Sugar intake (g/day), mean (SD)	43.44 (23.38)	51.61 (28.62)	.02	45.21 (26.17)	51.24 (28.29)	.02
Thiamin (mg/day), mean (SD)	0.96 (0.29)	0.92 (0.23)	.36	0.88 (0.20)	0.92 (0.23)	.03
Energy (kcal/day), mean (SD)	1849.44 (602.97)	1724.20 (574.52)	.17	1747.76 (595.26)	1736.33 (578.40)	.84
Triglyceride (mg/dL), mean (SD)	124.04 (65.34)	116.89 (77.77)	.46	119.24 (65.68)	117.76 (74.28)	.82
Vitamin A (μg RE/day), mean (SD)	420.96 (163.99)	445.56 (220.24)	.32	419.18 (216.12)	450.51 (226.26)	.14
Vitamin C (mg/day), mean (SD)	60.60 (32.77)	68.22 (39.52)	.12	61.14 (35.31)	68.57 (39.15)	.03
Vitamin D (μg/day), mean (SD)	5.15 (5.65)	4.79 (4.09)	.67	4.04 (3.35)	4.79 (4.06)	.02
Vitamin E (mg/day), mean (SD)	6.07 (3.42)	6.66 (3.14)	.24	6.46 (3.29)	6.65 (3.10)	.57
Zinc (mg/day), mean (SD)	5.16 (2.00)	5.14 (1.88)	.94	4.83 (1.66)	5.17 (1.87)	.04

[a] Values are reported as means (standard deviations. *P*< .05 was considered as statistically significant.
[b] AST: aspartate aminotransferase.
[c] BMI: body mass index.
[d] DBP: diastolic blood pressure.
[e] GGT: gamma-glutamyl transferase.
[f] HDL: high density lipoprotein.
[g] LDL: low density lipoprotein.
[h] MUFA: monounsaturated fatty acids.
[i] PUFA: polyunsaturated fatty acids.
[j] SBP: systolic blood pressure.
[k] SFA: saturated fatty acids.

Table S3. Descriptive statistics of participants excluded due to missing data - categorical variables. [a]

Characteristics	Case (n=58)	Control (n=3193)	*P*
Gender, n (%)			.002
Male	32 (55.2)	1100 (34.5)	
Female	26 (44.8)	2093 (65.5)	
Alcohol consumer, n (%)			<.001
No	17 (29.3)	1214 (38.0)	
Yes	40 (67.0)	1977 (61.9)	
Missing	1 (1.7)	2 (0.1)	
Smoker, n (%)			.002
No	29 (50.0)	2151 (67.4)	
Yes	28 (48.3)	1035 (32.4)	
Missing	1 (1.7)	7 (0.2)	
Married or cohabitating, n (%)			.76
No	7 (12.1)	481 (15.1)	
Yes	48 (82.8)	2515 (78.8)	
Missing	3 (5.2)	197 (6.2)	
Unemployed			.32
No	23 (39.7)	1578 (49.4)	
Yes	30 (51.7)	1355 (42.4)	
Missing	5 (8.6)	260 (8.1)	
Higher education, n (%)			.67
No	27 (46.6)	1431 (44.8)	
Yes	18 (31.0)	1155 (36.2)	
Missing	13 (22.4)	607 (19.0)	
Lower income, n (%)			.16
No	23 (39.7)	1418 (44.4)	
Yes	11 (19.0)	352 (11.0)	
Missing	24 (41.4)	1423 (44.6)	

[a] Values are reported as frequencies and proportion. $P < .05$ was considered as statistically significant.

Table S4. Descriptive statistics of participants excluded due to missing data - continuous variables. [a]

Characteristic	Case (n=58)	Control (n=3193)	*P*	Missing, n (%)
Age (years), mean (SD)	57.22 (7.91)	53.57 (8.94)	.001	0 (0.0)
AST (U/L), mean (SD)	23.85 (11.38)	23.37 (12.44)	.76	183 (5.6)
BMI (kg/m^2), mean (SD)	23.92 (2.75)	23.81 (3.09)	.78	196 (6.0)
Calcium (mg/day), mean (SD)	389.94 (139.21)	473.07 (215.06)	<.001	0 (0.0)
Carbohydrate (g/day), mean (SD)	311.52 (24.81)	300.62 (34.50)	.002	0 (0.0)
Carotene (μg/day), mean (SD)	1838.23 (936.73)	2026.46 (1180.47)	.14	0 (0.0)
Cholesterol (mg/day), mean (SD)	100.60 (41.61)	133.21 (82.95)	<.001	0 (0.0)
DBP (mmHg), mean (SD)	81.58 (8.72)	76.74 (10.70)	<.001	194 (56.0)
Fasting blood glucose (mg/dL), mean (SD)	99.42 (19.26)	95.81 (18.84)	.17	182 (5.7)
Fat (g/day), mean (SD)	25.82 (8.34)	29.47 (11.72)	.002	0 (0.0)
Fiber (g/day), mean (SD)	14.43 (6.73)	16.04 (7.34)	.08	0 (0.0)
GGT (IU/L), mean (SD)	35.58 (46.18)	28.48 (30.10)	.26	188 (5.8)
HDL (mg/dL), mean (SD)	58.38 (12.39)	58.92 (14.71)	.83	1869 (57.5)
Iron (mg/day), mean (SD)	9.29 (2.21)	9.95 (2.88)	.03	0 (0.0)
LDL (mg/dL), mean (SD)	125.87 (31.66)	118.53 (34.44)	.27	1869 (57.5)
Magnesium (mg/day), mean (SD)	150.33 (50.33)	170.45 (67.43)	.004	0 (0.0)
MUFA (g/day), mean (SD)	6.66 (2.78)	7.82 (3.91)	.003	0 (0.0)
Niacin (mg/day), mean (SD)	9.10 (2.07)	9.84 (2.56)	.009	0 (0.0)
Phosphorus (mg/day), mean (SD)	787.80 (158.20)	869.84 (221.28)	<.001	0 (0.0)
Potassium (mg/day), mean (SD)	2165.64 (693.11)	2362.92 (795.07)	.04	0 (0.0)
Protein (g/day), mean (SD)	57.24 (9.77)	60.34 (11.46)	.02	0 (0.0)
PUFA (g/day), mean (SD)	4.13 (1.52)	4.67 (2.06)	.01	0 (0.0)
SBP (mmHg), mean (SD)	132.54 (13.78)	126.20 (15.24)	.002	194 (6.0)
SFA (g/day), mean (SD)	7.26 (2.89)	8.83 (4.30)	<.001	0 (0.0)
Sodium (mg/day), mean (SD)	1743.51 (660.86)	1898.42 (807.25)	.08	0 (0.0)
Sugar intake (g/day), mean (SD)	44.97 (30.72)	49.52 (27.91)	.27	0 (0.0)
Thiamin (mg/day), mean (SD)	0.86 (0.25)	0.88 (0.23)	.60	0 (0.0)
Energy (kcal/day), mean (SD)	1594.90 (478.46)	1665.60 (582.87)	.27	0 (0.0)
Triglyceride (mg/dL), mean (SD)	105.67 (35.47)	120.65 (76.90)	.06	1869 (57.5)
Vitamin A (μg RE/day), mean (SD)	366.62 (163.53)	433.05 (216.11)	.003	0 (0.0)
Vitamin C (mg/day), mean (SD)	60.23 (39.75)	66.10 (38.70)	.27	0 (0.0)
Vitamin D (μg/day), mean (SD)	3.50 (2.42)	5.00 (4.43)	<.001	1 (0.03)
Vitamin E (mg/day), mean (SD)	5.74 (2.63)	6.44 (3.21)	.05	0 (0.0)
Zinc (mg/day), mean (SD)	4.41 (1.29)	4.98 (1.86)	.002	0 (0.0)

[a] Values are reported as means (standard deviations. *P*< .05 was considered as statistically significant.

Table S5. Model tuning results optimizing area under the curve (AUC) metrics. [a]

Model	AUC	Parameters	Predictors
LR			
SMOTE	0.71	{"solver": "saga", "penalty": "l2", "C": 0.1}	age, BMI, DBP, AST, protein, fat, carbohydrates, sugar, fiber, SFA, MUFA, PUFA, cholesterol, vitamin D, magnesium, iron, vitamin E, phosphorus, vitamin A, carotene
SMOTE+ENN	0.72	{"solver": "liblinear", "penalty": "l1", "C": 0.05}	age, DBP, protein, fat, carbohydrates, sugar, fiber, SFA, MUFA, PUFA, cholesterol, vitamin D, magnesium, iron, vitamin E, phosphorus, vitamin A, carotene, calcium, niacin
ADASYN	0.72	{"solver": "saga", "penalty": "l2", "C": 0.1}	age, BMI, DBP, AST, protein, fat, carbohydrates, sugar, fiber, SFA, MUFA, PUFA, cholesterol, vitamin D, magnesium, iron, vitamin E, phosphorus, vitamin A, carotene
PCUSTe-1	0.74	{"solver": "saga", "penalty": "elasticnet", "l1_ratio": 0.1, "C": 0.1}	age, SBP, DBP, triglycerides, HDL, AST, protein, fat, carbohydrates, fiber, SFA, PUFA, cholesterol, magnesium, iron, vitamin E, phosphorus, potassium, vitamin A, calcium
PCUSTe-2	0.72	{"solver": "liblinear", "penalty": "l1", "C": 0.1}	age, DBP, triglycerides, protein, fat, carbohydrates, sugar, fiber, SFA, MUFA, cholesterol, vitamin D, magnesium, iron, vitamin E, phosphorus, vitamin A, carotene, sodium, niacin
RF			
SMOTE	0.68	{"min_samples_split": 7, "min_samples_leaf": 8, "max_features": 0.001}	age, BMI, SBP, DBP, triglycerides, HDL, LDL, glucose, AST, GGT, energy, protein, sugar, fiber, MUFA, cholesterol, vitamin D, carotene, calcium, thiamin
SMOTE+ENN	0.67	{"min_samples_split": 8, "min_samples_leaf": 9, "max_features": 1e-06}	age, BMI, SBP, DBP, triglycerides, HDL, LDL, glucose, AST, GGT, energy, sugar, fiber, MUFA, cholesterol, vitamin D, vitamin E, potassium, calcium, thiamin
ADASYN	0.68	{"min_samples_split": 7, "min_samples_leaf": 8, "max_features": 0.001}	age, BMI, SBP, DBP, triglycerides, HDL, LDL, glucose, AST, GGT, energy, protein, sugar, fiber, MUFA, cholesterol, vitamin D, carotene, calcium, thiamin
PCUSTe-1	0.69	{"min_samples_split": 8, "min_samples_leaf": 9, "max_features": 1e-06}	age, BMI, SBP, DBP, HDL, LDL, AST, GGT, protein, sugar, SFA, MUFA, cholesterol, vitamin D, iron, vitamin E, calcium, vitamin C, niacin, thiamin
PCUSTe-2	0.65	{"min_samples_split": 3, "min_samples_leaf": 4, "max_features": 0.0001}	age, BMI, SBP, DBP, triglycerides, LDL, AST, energy, carbohydrates, sugar, fiber, MUFA, cholesterol, vitamin D, iron, vitamin E, sodium, calcium, vitamin C, niacin
SGD			
SMOTE	0.71	{"eta0": 0.001, "alpha": 0.1}	age, DBP, triglycerides, HDL, AST, protein, fat, carbohydrates, sugar, fiber, MUFA, PUFA, cholesterol, vitamin D, magnesium, iron, vitamin E, phosphorus, vitamin A, carotene

SMOTE+ENN	0.71	{"eta0": 0.001, "alpha": 0.1}	age, BMI, DBP, triglycerides, protein, fat, carbohydrates, sugar, fiber, SFA, MUFA, PUFA, cholesterol, vitamin D, magnesium, iron, vitamin A, carotene, calcium, niacin
ADASYN	0.71	{"eta0": 0.001, "alpha": 0.1}	age, BMI, DBP, protein, fat, carbohydrates, sugar, fiber, SFA, MUFA, PUFA, cholesterol, vitamin D, magnesium, iron, phosphorus, vitamin A, carotene, calcium, niacin
PCUSTe-1	0.71	{"eta0": 0.001, "alpha": 0.0001}	DBP, HDL, AST, energy, protein, carbohydrates, sugar, fiber, SFA, MUFA, PUFA, cholesterol, vitamin D, magnesium, zinc, vitamin E, potassium, vitamin A, carotene, thiamin
PCUSTe-2	0.71	{"eta0": 0.001, "alpha": 0.0001}	age, DBP, triglycerides, LDL, GGT, energy, protein, fat, carbohydrates, fiber, SFA, MUFA, PUFA, cholesterol, vitamin D, magnesium, phosphorus, vitamin A, sodium, thiamin
XGBoost			
SMOTE	0.67	{"subsample": 0.6, "reg_lambda": 10, "reg_alpha": 0, "n_estimators": 700, "min_child_weight": 5, "max_depth": 3, "learning_rate": 0.01, "gamma": 5, "colsample_bytree": 0.5}	age, BMI, SBP, DBP, triglycerides, HDL, LDL, AST, GGT, energy, protein, sugar, fiber, cholesterol, vitamin D, iron, vitamin E, carotene, vitamin C, thiamin
SMOTE+ENN	0.67	{"subsample": 0.6, "reg_lambda": 10, "reg_alpha": 0, "n_estimators": 700, "min_child_weight": 5, "max_depth": 3, "learning_rate": 0.01, "gamma": 5, "colsample_bytree": 0.5}	age, BMI, SBP, DBP, triglycerides, HDL, LDL, AST, GGT, energy, protein, carbohydrates, sugar, fiber, cholesterol, magnesium, vitamin E, phosphorus, vitamin C, thiamin
ADASYN	0.66	{"subsample": 0.6, "reg_lambda": 10, "reg_alpha": 0, "n_estimators": 700, "min_child_weight": 5, "max_depth": 3, "learning_rate": 0.01,	age, BMI, SBP, DBP, triglycerides, HDL, LDL, AST, GGT, energy, protein, carbohydrates, sugar, fiber, MUFA, cholesterol, vitamin D, carotene, vitamin C, thiamin

		"gamma": 5, "colsample_bytree": 0.5}	
PCUSTe-1	0.69	{"subsample": 0.6, "reg_lambda": 10, "reg_alpha": 0, "n_estimators": 700, "min_child_weight": 7, "max_depth": 4, "learning_rate": 0.05, "gamma": 5, "colsample_bytree": 0.9}	age, BMI, SBP, DBP, triglycerides, HDL, glucose, AST, GGT, protein, sugar, MUFA, vitamin D, magnesium, vitamin E, vitamin A, sodium, vitamin C, niacin, thiamin
PCUSTe-2	0.65	{"subsample": 0.6, "reg_lambda": 1, "reg_alpha": 0.01, "n_estimators": 700, "min_child_weight": 3, "max_depth": 3, "learning_rate": 0.01, "gamma": 5, "colsample_bytree": 0.9}	age, BMI, DBP, triglycerides, glucose, AST, energy, protein, fat, sugar, fiber, MUFA, cholesterol, vitamin D, magnesium, iron, carotene, sodium, calcium, niacin
SVM			
SMOTE	0.72	{"kernel": "linear", "gamma": 1.0, "coef0": 0.001, "C": 0.001}	age, BMI, DBP, protein, fat, carbohydrates, sugar, fiber, SFA, MUFA, PUFA, cholesterol, vitamin D, magnesium, iron, phosphorus, vitamin A, carotene, calcium, niacin
SMOTE+ENN	0.70	{"kernel": "linear", "gamma": 1.0, "coef0": 0.001, "C": 0.001}	age, BMI, DBP, triglycerides, protein, fat, carbohydrates, sugar, fiber, SFA, MUFA, PUFA, cholesterol, vitamin D, magnesium, iron, vitamin A, carotene, calcium, niacin
ADASYN	0.71	{"kernel": "linear", "gamma": 1.0, "coef0": 0.001, "C": 0.001}	age, BMI, DBP, protein, fat, carbohydrates, sugar, fiber, SFA, MUFA, PUFA, cholesterol, vitamin D, magnesium, iron, phosphorus, vitamin A, carotene, calcium, niacin
PCUSTe-1	0.68	{"kernel": "linear", "gamma": 0.0001, "coef0": 1e-06, "C": 0.1}	age, SBP, DBP, triglycerides, HDL, protein, carbohydrates, fiber, SFA, MUFA, PUFA, cholesterol, vitamin D, iron, vitamin E, phosphorus, potassium, vitamin A, calcium, niacin
PCUSTe-2	0.70	{"kernel": "linear", "gamma": 0.0001, "coef0": 1e-06, "C": 0.1}	age, BMI, SBP, triglycerides, AST, protein, fat, carbohydrates, sugar, fiber, MUFA, cholesterol, vitamin D, magnesium, iron, phosphorus, vitamin A, sodium, calcium, thiamin

[a] Hyperparameters were tuned exclusively on training split via RandomizedSearchCV using 20 predictors selected using RFE (random feature elimination) within same internal cross-validation pipeline. Reported AUC score is derived from test fold within this cross-validation.

Table S6. Model tuning results optimizing Matthews correlation coefficient (MCC) metric. [a]

Model	MCC	Parameters	Predictors
LR			
SMOTE	0.10	{"solver": "saga", "penalty": "elasticnet", "l1_ratio": 0.5, "C": 0.5}	age, BMI, DBP, AST, protein, fat, carbohydrates, sugar, fiber, SFA, MUFA, PUFA, cholesterol, vitamin D, magnesium, iron, vitamin E, phosphorus, vitamin A, carotene
SMOTE+ENN	0.10	{"solver": "liblinear", "penalty": "l1", "C": 0.05}	age, DBP, protein, fat, carbohydrates, sugar, fiber, SFA, MUFA, PUFA, cholesterol, vitamin D, magnesium, iron, vitamin E, phosphorus, vitamin A, carotene, calcium, niacin
ADASYN	0.10	{"solver": "saga", "penalty": "l2", "C": 0.1}	age, BMI, DBP, AST, protein, fat, carbohydrates, sugar, fiber, SFA, MUFA, PUFA, cholesterol, vitamin D, magnesium, iron, vitamin E, phosphorus, vitamin A, carotene
PCUSTe-1	0.10	{"solver": "saga", "penalty": "l1", "C": 0.5}	age, SBP, DBP, triglycerides, HDL, AST, protein, fat, carbohydrates, fiber, SFA, PUFA, cholesterol, magnesium, iron, vitamin E, phosphorus, potassium, vitamin A, calcium
PCUSTe-2	0.10	{"solver": "saga", "penalty": "elasticnet", "l1_ratio": 0.1, "C": 0.5}	age, DBP, triglycerides, protein, fat, carbohydrates, sugar, fiber, SFA, MUFA, cholesterol, vitamin D, magnesium, iron, vitamin E, phosphorus, vitamin A, carotene, sodium, niacin
RF			
SMOTE	0.06	{"min_samples_split": 8, "min_samples_leaf": 9, "max_features": 1e-06}	age, BMI, SBP, DBP, triglycerides, HDL, LDL, glucose, AST, GGT, energy, protein, sugar, fiber, MUFA, cholesterol, vitamin D, carotene, calcium, thiamin
SMOTE+ENN	0.09	{"min_samples_split": 9, "min_samples_leaf": 10, "max_features": 0.001}	age, BMI, SBP, DBP, triglycerides, HDL, LDL, glucose, AST, GGT, energy, sugar, fiber, MUFA, cholesterol, vitamin D, vitamin E, potassium, calcium, thiamin
ADASYN	0.07	{"min_samples_split": 8, "min_samples_leaf": 2, "max_features": 0.0001}	age, BMI, SBP, DBP, triglycerides, HDL, LDL, glucose, AST, GGT, energy, protein, sugar, fiber, MUFA, cholesterol, vitamin D, carotene, calcium, thiamin
PCUSTe-1	0.09	{"min_samples_split": 3, "min_samples_leaf": 4, "max_features": 0.0001}	age, BMI, SBP, DBP, HDL, LDL, AST, GGT, protein, sugar, SFA, MUFA, cholesterol, vitamin D, iron, vitamin E, calcium, vitamin C, niacin, thiamin
PCUSTe-2	0.07	{"min_samples_split": 10, "min_samples_leaf": 5, "max_features": 0.001}	age, BMI, SBP, DBP, triglycerides, LDL, AST, energy, carbohydrates, sugar, fiber, MUFA, cholesterol, vitamin D, iron, vitamin E, sodium, calcium, vitamin C, niacin
SGD			
SMOTE	0.10	{"eta0": 0.001, "alpha": 0.001}	age, DBP, triglycerides, HDL, AST, protein, fat, carbohydrates, sugar, fiber, MUFA, PUFA, cholesterol, vitamin D, magnesium, iron, vitamin E, phosphorus, vitamin A, carotene

SMOTE+ENN	0.09	{"eta0": 0.001, "alpha": 0.01}	age, BMI, DBP, triglycerides, protein, fat, carbohydrates, sugar, fiber, SFA, MUFA, PUFA, cholesterol, vitamin D, magnesium, iron, vitamin A, carotene, calcium, niacin
ADASYN	0.09	{"eta0": 0.001, "alpha": 0.01}	age, BMI, DBP, protein, fat, carbohydrates, sugar, fiber, SFA, MUFA, PUFA, cholesterol, vitamin D, magnesium, iron, phosphorus, vitamin A, carotene, calcium, niacin
PCUSTe-1	0.08	{"eta0": 0.01, "alpha": 0.01}	DBP, HDL, AST, energy, protein, carbohydrates, sugar, fiber, SFA, MUFA, PUFA, cholesterol, vitamin D, magnesium, zinc, vitamin E, potassium, vitamin A, carotene, thiamin
PCUSTe-2	0.09	{"eta0": 0.001, "alpha": 0.01}	age, DBP, triglycerides, LDL, GGT, energy, protein, fat, carbohydrates, fiber, SFA, MUFA, PUFA, cholesterol, vitamin D, magnesium, phosphorus, vitamin A, sodium, thiamin
XGBoost			
SMOTE	0.04	{"subsample": 1.0, "reg_lambda": 1, "reg_alpha": 1, "n_estimators": 700, "min_child_weight": 5, "max_depth": 3, "learning_rate": 0.2, "gamma": 0.1, "colsample_bytree": 0.9}	age, BMI, SBP, DBP, triglycerides, HDL, LDL, AST, GGT, energy, protein, sugar, fiber, cholesterol, vitamin D, iron, vitamin E, carotene, vitamin C, thiamin
SMOTE+ENN	0.08	{"subsample": 0.6, "reg_lambda": 1, "reg_alpha": 0.001, "n_estimators": 700, "min_child_weight": 3, "max_depth": 5, "learning_rate": 0.01, "gamma": 0, "colsample_bytree": 0.9}	age, BMI, SBP, DBP, triglycerides, HDL, LDL, AST, GGT, energy, protein, carbohydrates, sugar, fiber, cholesterol, magnesium, vitamin E, phosphorus, vitamin C, thiamin
ADASYN	0.08	{"subsample": 0.6, "reg_lambda": 1, "reg_alpha": 0, "n_estimators": 700, "min_child_weight": 3, "max_depth": 4, "learning_rate": 0.01,	age, BMI, SBP, DBP, triglycerides, HDL, LDL, AST, GGT, energy, protein, carbohydrates, sugar, fiber, MUFA, cholesterol, vitamin D, carotene, vitamin C, thiamin

			"gamma": 0, "colsample_bytree": 0.7}	
	PCUSTe-1	0.07	{"subsample": 0.6, "reg_lambda": 10, "reg_alpha": 0, "n_estimators": 700, "min_child_weight": 5, "max_depth": 3, "learning_rate": 0.01, "gamma": 5, "colsample_bytree": 0.5}	age, BMI, SBP, DBP, triglycerides, HDL, glucose, AST, GGT, protein, sugar, MUFA, vitamin D, magnesium, vitamin E, vitamin A, sodium, vitamin C, niacin, thiamin
	PCUSTe-2	0.09	{"subsample": 0.6, "reg_lambda": 1, "reg_alpha": 0.01, "n_estimators": 700, "min_child_weight": 3, "max_depth": 3, "learning_rate": 0.01, "gamma": 5, "colsample_bytree": 0.9}	age, BMI, DBP, triglycerides, glucose, AST, energy, protein, fat, sugar, fiber, MUFA, cholesterol, vitamin D, magnesium, iron, carotene, sodium, calcium, niacin
SVM				
	SMOTE	0.10	{"kernel": "rbf", "gamma": 1e-06, "coef0": 1e-06, "C": 100.0}	age, BMI, DBP, protein, fat, carbohydrates, sugar, fiber, SFA, MUFA, PUFA, cholesterol, vitamin D, magnesium, iron, phosphorus, vitamin A, carotene, calcium, niacin
	SMOTE+ENN	0.08	{"kernel": "linear", "gamma": 1.0, "coef0": 0.001, "C": 0.001}	age, BMI, DBP, triglycerides, protein, fat, carbohydrates, sugar, fiber, SFA, MUFA, PUFA, cholesterol, vitamin D, magnesium, iron, vitamin A, carotene, calcium, niacin
	ADASYN	0.09	{"kernel": "rbf", "gamma": 1e-06, "coef0": 1e-06, "C": 100.0}	age, BMI, DBP, protein, fat, carbohydrates, sugar, fiber, SFA, MUFA, PUFA, cholesterol, vitamin D, magnesium, iron, phosphorus, vitamin A, carotene, calcium, niacin
	PCUSTe-1	0.07	{"kernel": "linear", "gamma": 0.0001, "coef0": 1e-06, "C": 0.1}	age, SBP, DBP, triglycerides, HDL, protein, carbohydrates, fiber, SFA, MUFA, PUFA, cholesterol, vitamin D, iron, vitamin E, phosphorus, potassium, vitamin A, calcium, niacin
	PCUSTe-2	0.09	{"kernel": "linear", "gamma": 0.0001, "coef0": 1e-06, "C": 0.1}	age, BMI, SBP, triglycerides, AST, protein, fat, carbohydrates, sugar, fiber, MUFA, cholesterol, vitamin D, magnesium, iron, phosphorus, vitamin A, sodium, calcium, thiamin

[a] Hyperparameters were tuned exclusively on training split via RandomizedSearchCV using 20 predictors selected using RFE (random feature elimination) within same internal cross-validation pipeline. Reported MCC score is derived from test fold within this cross-validation pipeline.

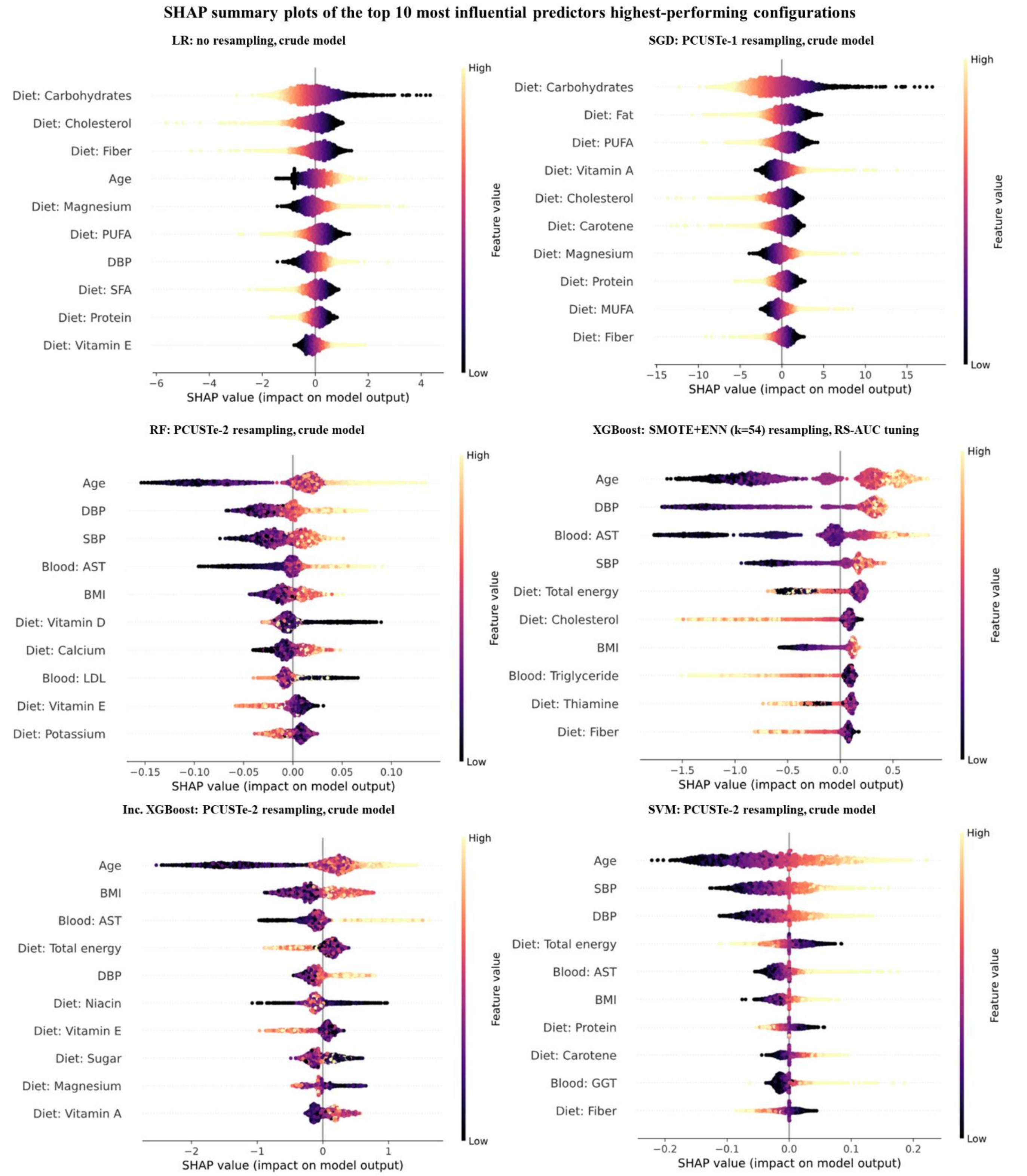

Figure S1. SHAP summary plots for the top 10 most influential predictors in the final model configurations across six ML algorithms. Features are ranked by mean absolute SHAP value within each model, with only the top 10 predictors shown. Each point represents an individual observation, colored by the corresponding feature value (low to high). Positive SHAP values indicate increased predicted GI cancer risk.

Table S7. Model evaluation results without hyperparameter tuning, using all predictors. [a]

Model	k [b]	Point estimate on test split (95% CI)			
		Sensitivity	Specificity	AUC	MCC
LR					
No resampling		0.70 (0.57–0.83)	0.71 (0.69–0.72)	0.75 (0.68–0.82)	0.13 (0.08–0.17)
ADASYN	5	0.64 (0.50–0.77)	0.71 (0.70–0.73)	0.74 (0.67–0.80)	0.11 (0.06–0.16)
SMOTE	5	0.64 (0.50–0.77)	0.72 (0.70–0.74)	0.74 (0.68–0.81)	0.11 (0.06–0.16)
SMOTE+ENN	5	0.75 (0.63–0.88)	0.62 (0.60–0.64)	0.74 (0.67–0.81)	0.11 (0.07–0.15)
PCUSTe-1		0.66 (0.53–0.80)	0.67 (0.65–0.69)	0.72 (0.65–0.79)	0.10 (0.06–0.14)
PCUSTe-2		0.62 (0.47–0.76)	0.66 (0.64–0.68)	0.68 (0.61–0.76)	0.08 (0.04–0.13)
RF					
No resampling		0.60 (0.45–0.73)	0.66 (0.64–0.68)	0.69 (0.60–0.77)	0.08 (0.03–0.12)
ADASYN	5	0.04 (0.00–0.10)	0.99 (0.99–0.99)	0.68 (0.59–0.75)	0.04 (-0.02–0.13)
SMOTE	5	0.04 (0.00–0.11)	0.99 (0.98–0.99)	0.69 (0.61–0.76)	0.04 (-0.02–0.12)
SMOTE+ENN	5	0.19 (0.09–0.30)	0.96 (0.95–0.96)	0.70 (0.62–0.77)	0.10 (0.03–0.17)
PCUSTe-1		0.66 (0.52–0.79)	0.63 (0.61–0.65)	0.71 (0.64–0.78)	0.09 (0.04–0.12)
PCUSTe-2		0.77 (0.65–0.89)	0.62 (0.60–0.64)	0.73 (0.65–0.81)	0.11 (0.07–0.15)
SGD					
No resampling		0.23 (0.12–0.36)	0.89 (0.87–0.90)	0.64 (0.55–0.71)	0.05 (0.00–0.11)
ADASYN	5	0.49 (0.34–0.64)	0.77 (0.75–0.79)	0.70 (0.62–0.78)	0.09 (0.04–0.14)
SMOTE	5	0.60 (0.45–0.73)	0.67 (0.65–0.69)	0.67 (0.58–0.75)	0.08 (0.04–0.13)
SMOTE+ENN	5	0.58 (0.44–0.72)	0.65 (0.63–0.67)	0.66 (0.58–0.74)	0.07 (0.03–0.11)
PCUSTe-1		0.77 (0.64–0.89)	0.65 (0.63–0.67)	0.77 (0.70–0.84)	0.12 (0.08–0.16)
PCUSTe-2		0.64 (0.50–0.78)	0.67 (0.65–0.69)	0.72 (0.64–0.79)	0.09 (0.05–0.14)
SVM					
No resampling		0.64 (0.50–0.77)	0.54 (0.52–0.56)	0.67 (0.58–0.75)	0.05 (0.01–0.09)
ADASYN	5	0.13 (0.04–0.23)	0.99 (0.98–0.99)	0.59 (0.50–0.68)	0.12 (0.03–0.24)
SMOTE	5	0.13 (0.04–0.23)	0.98 (0.98–0.99)	0.58 (0.49–0.67)	0.12 (0.03–0.22)
SMOTE+ENN	5	0.17 (0.07–0.29)	0.96 (0.95–0.97)	0.60 (0.51–0.68)	0.09 (0.02–0.18)
PCUSTe-1		0.75 (0.63–0.86)	0.59 (0.57–0.61)	0.74 (0.68–0.80)	0.10 (0.06–0.14)
PCUSTe-2		0.79 (0.67–0.90)	0.60 (0.58–0.62)	0.74 (0.67–0.80)	0.11 (0.07–0.15)
XGBoost					
No resampling		0.06 (0.00–0.14)	0.98 (0.97–0.98)	0.67 (0.60–0.75)	0.04 (-0.02–0.10)
ADASYN	5	0.02 (0.00–0.07)	0.99 (0.99–1.00)	0.65 (0.56–0.73)	0.03 (-0.01–0.11)
SMOTE	5	0.04 (0.00–0.12)	0.99 (0.99–0.99)	0.64 (0.55–0.72)	0.05 (-0.02–0.15)
SMOTE+ENN	5	0.24 (0.12–0.35)	0.96 (0.95–0.97)	0.70 (0.63–0.77)	0.14 (0.06–0.22)
PCUSTe-1		0.64 (0.50–0.77)	0.62 (0.60–0.64)	0.68 (0.61–0.75)	0.07 (0.03–0.11)
PCUSTe-2		0.66 (0.52–0.80)	0.61 (0.59–0.63)	0.68 (0.60–0.75)	0.08 (0.04–0.12)
Inc. XGBoost					
No resampling		0.42 (0.28–0.57)	0.83 (0.82–0.85)	0.70 (0.63–0.77)	0.10 (0.04–0.16)
ADASYN	5	0.23 (0.12–0.36)	0.92 (0.91–0.93)	0.66 (0.58–0.73)	0.08 (0.02–0.15)
SMOTE	5	0.26 (0.14–0.38)	0.92 (0.91–0.93)	0.68 (0.59–0.75)	0.09 (0.03–0.16)
SMOTE+ENN	5	0.38 (0.25–0.52)	0.86 (0.85–0.87)	0.70 (0.63–0.77)	0.10 (0.04–0.15)
PCUSTe-1		0.56 (0.41–0.70)	0.62 (0.60–0.64)	0.68 (0.61–0.75)	0.05 (0.01–0.09)
PCUSTe-2		0.70 (0.57–0.83)	0.62 (0.60–0.64)	0.68 (0.61–0.76)	0.09 (0.05–0.13)

[a] Values represent point estimates on test set with 95% CIs calculated by bootstrapping test set.
[b] k: k-nearest neighbors parameter used in oversampling methods. In crude evaluation, default value was used (k=5).

Table S8. Model evaluation results with hyperparameter tuning optimizing area under the curve (AUC) metric, using 20 most predictors selected with RFE. [a]

Model	k	Point estimate on test split (95% CI)			
		Sensitivity	Specificity	AUC	MCC
LR					
ADASYN	2	0.66 (0.53–0.79)	0.70 (0.68–0.72)	0.73 (0.66–0.80)	0.11 (0.07–0.15)
ADASYN	5	0.66 (0.53–0.79)	0.70 (0.68–0.71)	0.74 (0.67–0.80)	0.11 (0.06–0.15)
ADASYN	10	0.66 (0.53–0.79)	0.69 (0.67–0.71)	0.73 (0.66–0.81)	0.11 (0.06–0.15)
ADASYN	32	0.68 (0.55–0.81)	0.70 (0.68–0.72)	0.73 (0.66–0.81)	0.12 (0.07–0.16)
ADASYN	54	0.70 (0.57–0.83)	0.70 (0.68–0.72)	0.74 (0.67–0.81)	0.12 (0.08–0.17)
ADASYN	87	0.70 (0.57–0.83)	0.70 (0.68–0.72)	0.73 (0.66–0.81)	0.12 (0.08–0.17)
SMOTE	2	0.66 (0.53–0.79)	0.70 (0.68–0.72)	0.73 (0.67–0.80)	0.11 (0.06–0.15)
SMOTE	5	0.66 (0.53–0.79)	0.70 (0.68–0.72)	0.74 (0.67–0.81)	0.11 (0.07–0.15)
SMOTE	10	0.66 (0.53–0.79)	0.70 (0.68–0.72)	0.73 (0.66–0.80)	0.11 (0.07–0.15)
SMOTE	32	0.64 (0.50–0.78)	0.70 (0.69–0.72)	0.73 (0.66–0.80)	0.11 (0.06–0.15)
SMOTE	54	0.72 (0.60–0.85)	0.70 (0.68–0.72)	0.74 (0.67–0.81)	0.13 (0.09–0.17)
SMOTE	87	0.68 (0.55–0.81)	0.70 (0.68–0.72)	0.73 (0.66–0.81)	0.12 (0.08–0.16)
SMOTE+ENN	2	0.68 (0.56–0.81)	0.59 (0.57–0.61)	0.72 (0.65–0.79)	0.08 (0.04–0.12)
SMOTE+ENN	5	0.70 (0.57–0.84)	0.56 (0.54–0.58)	0.72 (0.65–0.79)	0.08 (0.04–0.11)
SMOTE+ENN	10	0.77 (0.66–0.89)	0.54 (0.52–0.56)	0.72 (0.65–0.78)	0.09 (0.05–0.12)
SMOTE+ENN	32	0.79 (0.68–0.91)	0.50 (0.48–0.52)	0.71 (0.64–0.78)	0.08 (0.05–0.12)
SMOTE+ENN	54	0.77 (0.65–0.89)	0.48 (0.46–0.50)	0.71 (0.64–0.78)	0.07 (0.04–0.10)
SMOTE+ENN	87	0.79 (0.68–0.91)	0.47 (0.45–0.49)	0.71 (0.63–0.78)	0.07 (0.04–0.11)
PCUSTe-1		0.71 (0.58–0.83)	0.67 (0.65–0.69)	0.74 (0.68–0.81)	0.11 (0.07–0.15)
PCUSTe-2		0.84 (0.72–0.93)	0.51 (0.49–0.53)	0.73 (0.66–0.79)	0.10 (0.06–0.13)
RF					
ADASYN	2	0.09 (0.02–0.17)	0.99 (0.99–0.99)	0.69 (0.61–0.77)	0.11 (0.02–0.22)
ADASYN	5	0.13 (0.04–0.23)	0.98 (0.98–0.99)	0.70 (0.63–0.78)	0.11 (0.02–0.20)
ADASYN	10	0.19 (0.09–0.30)	0.96 (0.96–0.97)	0.70 (0.62–0.77)	0.12 (0.04–0.19)
ADASYN	32	0.21 (0.10–0.32)	0.94 (0.93–0.95)	0.68 (0.61–0.76)	0.09 (0.03–0.16)
ADASYN	54	0.26 (0.15–0.38)	0.93 (0.92–0.94)	0.70 (0.62–0.77)	0.10 (0.04–0.17)
ADASYN	87	0.21 (0.11–0.33)	0.93 (0.92–0.94)	0.69 (0.61–0.77)	0.08 (0.02–0.14)
SMOTE	2	0.02 (0.00–0.07)	0.99 (0.98–0.99)	0.71 (0.63–0.79)	0.02 (-0.02–0.09)
SMOTE	5	0.15 (0.06–0.25)	0.98 (0.97–0.98)	0.72 (0.64–0.79)	0.12 (0.03–0.21)
SMOTE	10	0.17 (0.07–0.27)	0.96 (0.95–0.97)	0.68 (0.60–0.76)	0.10 (0.03–0.17)
SMOTE	32	0.21 (0.11–0.32)	0.95 (0.94–0.96)	0.70 (0.62–0.77)	0.10 (0.03–0.16)
SMOTE	54	0.28 (0.16–0.39)	0.94 (0.93–0.95)	0.70 (0.62–0.77)	0.12 (0.05–0.19)
SMOTE	87	0.28 (0.15–0.40)	0.93 (0.92–0.94)	0.70 (0.63–0.78)	0.11 (0.05–0.18)
SMOTE+ENN	2	0.22 (0.11–0.33)	0.96 (0.95–0.97)	0.71 (0.64–0.78)	0.13 (0.05–0.20)
SMOTE+ENN	5	0.24 (0.12–0.35)	0.92 (0.91–0.93)	0.70 (0.62–0.77)	0.08 (0.02–0.14)
SMOTE+ENN	10	0.36 (0.22–0.51)	0.87 (0.85–0.88)	0.69 (0.61–0.76)	0.10 (0.04–0.15)
SMOTE+ENN	32	0.49 (0.35–0.64)	0.78 (0.76–0.80)	0.69 (0.62–0.76)	0.09 (0.04–0.14)
SMOTE+ENN	54	0.49 (0.35–0.63)	0.74 (0.73–0.76)	0.67 (0.60–0.74)	0.08 (0.03–0.12)
SMOTE+ENN	87	0.54 (0.39–0.68)	0.71 (0.69–0.73)	0.68 (0.60–0.76)	0.08 (0.03–0.12)
PCUSTe-1		0.64 (0.51–0.77)	0.67 (0.65–0.69)	0.71 (0.64–0.77)	0.09 (0.05–0.13)

	PCUSTe-2		0.71 (0.57–0.83)	0.67 (0.65–0.69)	0.75 (0.69–0.81)	0.11 (0.07–0.16)
SGD						
	ADASYN	2	0.60 (0.46–0.74)	0.68 (0.66–0.70)	0.71 (0.64–0.78)	0.09 (0.04–0.13)
	ADASYN	5	0.60 (0.46–0.74)	0.68 (0.66–0.70)	0.71 (0.64–0.78)	0.08 (0.04–0.13)
	ADASYN	10	0.60 (0.45–0.73)	0.68 (0.66–0.70)	0.72 (0.65–0.78)	0.09 (0.04–0.13)
	ADASYN	32	0.60 (0.45–0.74)	0.68 (0.66–0.70)	0.71 (0.64–0.78)	0.09 (0.04–0.13)
	ADASYN	54	0.64 (0.50–0.77)	0.68 (0.66–0.70)	0.71 (0.65–0.78)	0.10 (0.05–0.14)
	ADASYN	87	0.62 (0.47–0.75)	0.68 (0.66–0.70)	0.71 (0.64–0.78)	0.09 (0.05–0.13)
	SMOTE	2	0.71 (0.58–0.83)	0.69 (0.68–0.71)	0.74 (0.67–0.81)	0.12 (0.08–0.16)
	SMOTE	5	0.70 (0.58–0.84)	0.70 (0.68–0.72)	0.74 (0.67–0.81)	0.12 (0.08–0.17)
	SMOTE	10	0.66 (0.52–0.81)	0.70 (0.68–0.72)	0.73 (0.66–0.80)	0.11 (0.06–0.15)
	SMOTE	32	0.68 (0.55–0.82)	0.70 (0.68–0.72)	0.74 (0.67–0.81)	0.12 (0.07–0.16)
	SMOTE	54	0.70 (0.58–0.84)	0.70 (0.68–0.72)	0.73 (0.65–0.80)	0.12 (0.08–0.17)
	SMOTE	87	0.70 (0.58–0.84)	0.70 (0.68–0.72)	0.74 (0.67–0.81)	0.12 (0.08–0.17)
	SMOTE+ENN	2	0.75 (0.63–0.87)	0.58 (0.56–0.60)	0.73 (0.66–0.79)	0.09 (0.06–0.13)
	SMOTE+ENN	5	0.77 (0.65–0.89)	0.53 (0.51–0.55)	0.73 (0.66–0.79)	0.08 (0.05–0.12)
	SMOTE+ENN	10	0.79 (0.68–0.91)	0.48 (0.46–0.50)	0.72 (0.65–0.78)	0.08 (0.04–0.11)
	SMOTE+ENN	32	0.85 (0.75–0.95)	0.42 (0.40–0.44)	0.72 (0.66–0.78)	0.08 (0.05–0.11)
	SMOTE+ENN	54	0.83 (0.72–0.94)	0.39 (0.37–0.41)	0.71 (0.64–0.78)	0.07 (0.03–0.10)
	SMOTE+ENN	87	0.85 (0.74–0.95)	0.38 (0.36–0.40)	0.71 (0.63–0.78)	0.07 (0.03–0.10)
	PCUSTe-1		0.56 (0.42–0.69)	0.70 (0.68–0.72)	0.67 (0.60–0.75)	0.08 (0.04–0.12)
	PCUSTe-2		0.64 (0.49–0.78)	0.71 (0.69–0.72)	0.71 (0.64–0.79)	0.11 (0.06–0.15)
SVM						
	ADASYN	2	0.64 (0.50–0.78)	0.68 (0.66–0.70)	0.72 (0.65–0.79)	0.10 (0.05–0.14)
	ADASYN	5	0.68 (0.54–0.81)	0.67 (0.65–0.69)	0.73 (0.66–0.79)	0.11 (0.06–0.15)
	ADASYN	10	0.68 (0.54–0.81)	0.68 (0.66–0.70)	0.73 (0.66–0.79)	0.11 (0.06–0.15)
	ADASYN	32	0.60 (0.45–0.74)	0.68 (0.66–0.70)	0.73 (0.66–0.79)	0.08 (0.04–0.13)
	ADASYN	54	0.64 (0.50–0.78)	0.68 (0.66–0.70)	0.73 (0.66–0.79)	0.10 (0.05–0.14)
	ADASYN	87	0.64 (0.50–0.78)	0.68 (0.66–0.70)	0.72 (0.66–0.79)	0.10 (0.05–0.14)
	SMOTE	2	0.64 (0.50–0.78)	0.68 (0.66–0.70)	0.72 (0.66–0.79)	0.10 (0.05–0.14)
	SMOTE	5	0.66 (0.52–0.80)	0.68 (0.66–0.70)	0.73 (0.66–0.79)	0.10 (0.06–0.14)
	SMOTE	10	0.68 (0.54–0.81)	0.68 (0.66–0.70)	0.73 (0.66–0.79)	0.11 (0.06–0.15)
	SMOTE	32	0.64 (0.50–0.77)	0.69 (0.67–0.71)	0.73 (0.66–0.79)	0.10 (0.05–0.14)
	SMOTE	54	0.60 (0.46–0.74)	0.68 (0.66–0.70)	0.73 (0.66–0.79)	0.08 (0.04–0.13)
	SMOTE	87	0.62 (0.47–0.75)	0.69 (0.66–0.70)	0.72 (0.66–0.79)	0.09 (0.05–0.14)
	SMOTE+ENN	2	0.73 (0.60–0.85)	0.62 (0.60–0.64)	0.74 (0.68–0.80)	0.10 (0.06–0.14)
	SMOTE+ENN	5	0.73 (0.60–0.85)	0.58 (0.56–0.60)	0.74 (0.68–0.80)	0.09 (0.05–0.13)
	SMOTE+ENN	10	0.75 (0.63–0.87)	0.55 (0.53–0.57)	0.73 (0.67–0.79)	0.08 (0.05–0.12)
	SMOTE+ENN	32	0.83 (0.71–0.94)	0.51 (0.49–0.53)	0.73 (0.67–0.79)	0.10 (0.06–0.13)
	SMOTE+ENN	54	0.81 (0.70–0.92)	0.48 (0.46–0.50)	0.72 (0.66–0.79)	0.08 (0.05–0.12)
	SMOTE+ENN	87	0.79 (0.68–0.91)	0.47 (0.45–0.49)	0.72 (0.66–0.79)	0.07 (0.04–0.11)
	PCUSTe-1		0.64 (0.50–0.78)	0.66 (0.64–0.68)	0.72 (0.65–0.79)	0.09 (0.05–0.13)
	PCUSTe-2		0.62 (0.49–0.76)	0.67 (0.65–0.69)	0.71 (0.63–0.79)	0.09 (0.04–0.13)
XGBoost						
	ADASYN	2	0.45 (0.31–0.59)	0.82 (0.80–0.83)	0.71 (0.64–0.78)	0.10 (0.04–0.15)
	ADASYN	5	0.47 (0.33–0.61)	0.80 (0.79–0.82)	0.72 (0.65–0.79)	0.10 (0.05–0.15)

ADASYN	10	0.45 (0.31–0.60)	0.80 (0.79–0.82)	0.72 (0.65–0.78)	0.09 (0.04–0.14)
ADASYN	32	0.49 (0.36–0.63)	0.80 (0.79–0.82)	0.71 (0.64–0.78)	0.10 (0.06–0.15)
ADASYN	54	0.45 (0.31–0.60)	0.81 (0.79–0.82)	0.72 (0.65–0.79)	0.09 (0.04–0.15)
ADASYN	87	0.47 (0.33–0.62)	0.80 (0.79–0.82)	0.71 (0.64–0.78)	0.10 (0.05–0.15)
SMOTE	2	0.47 (0.33–0.61)	0.82 (0.80–0.83)	0.72 (0.64–0.79)	0.10 (0.05–0.16)
SMOTE	5	0.51 (0.38–0.66)	0.81 (0.79–0.82)	0.73 (0.65–0.79)	0.11 (0.06–0.16)
SMOTE	10	0.51 (0.37–0.65)	0.81 (0.79–0.82)	0.72 (0.65–0.79)	0.11 (0.06–0.17)
SMOTE	32	0.47 (0.33–0.60)	0.81 (0.79–0.83)	0.72 (0.65–0.79)	0.10 (0.05–0.15)
SMOTE	54	0.47 (0.33–0.60)	0.80 (0.79–0.82)	0.72 (0.65–0.79)	0.10 (0.05–0.14)
SMOTE	87	0.47 (0.33–0.62)	0.81 (0.79–0.82)	0.72 (0.65–0.78)	0.10 (0.05–0.15)
SMOTE+ENN	2	0.51 (0.37–0.65)	0.76 (0.74–0.78)	0.72 (0.66–0.79)	0.09 (0.04–0.14)
SMOTE+ENN	5	0.60 (0.46–0.74)	0.72 (0.70–0.73)	0.72 (0.65–0.79)	0.10 (0.05–0.14)
SMOTE+ENN	10	0.64 (0.50–0.78)	0.69 (0.67–0.70)	0.72 (0.65–0.79)	0.10 (0.06–0.14)
SMOTE+ENN	32	0.70 (0.57–0.83)	0.63 (0.61–0.65)	0.72 (0.65–0.79)	0.10 (0.06–0.14)
SMOTE+ENN	54	0.77 (0.65–0.89)	0.60 (0.58–0.62)	0.73 (0.66–0.79)	0.11 (0.07–0.14)
SMOTE+ENN	87	0.75 (0.62–0.87)	0.60 (0.58–0.62)	0.73 (0.65–0.79)	0.10 (0.06–0.14)
PCUSTe-1		0.64 (0.50–0.77)	0.67 (0.65–0.68)	0.73 (0.67–0.79)	0.09 (0.05–0.13)
PCUSTe-2		0.73 (0.60–0.85)	0.63 (0.61–0.65)	0.72 (0.64–0.79)	0.10 (0.06–0.14)
Inc. XGBoost					
ADASYN	2	0.62 (0.48–0.75)	0.66 (0.64–0.67)	0.68 (0.61–0.75)	0.08 (0.04–0.12)
ADASYN	5	0.68 (0.55–0.82)	0.62 (0.60–0.64)	0.70 (0.63–0.78)	0.09 (0.05–0.13)
ADASYN	10	0.64 (0.50–0.77)	0.62 (0.60–0.63)	0.71 (0.64–0.78)	0.07 (0.03–0.11)
ADASYN	32	0.64 (0.51–0.78)	0.62 (0.60–0.64)	0.68 (0.61–0.76)	0.08 (0.04–0.12)
ADASYN	54	0.64 (0.51–0.78)	0.64 (0.62–0.66)	0.70 (0.64–0.78)	0.08 (0.04–0.12)
ADASYN	87	0.62 (0.48–0.76)	0.66 (0.64–0.68)	0.71 (0.65–0.78)	0.08 (0.04–0.13)
SMOTE	2	0.64 (0.51–0.77)	0.63 (0.62–0.65)	0.69 (0.61–0.76)	0.08 (0.04–0.12)
SMOTE	5	0.58 (0.44–0.72)	0.65 (0.63–0.67)	0.69 (0.62–0.76)	0.07 (0.02–0.11)
SMOTE	10	0.68 (0.54–0.82)	0.61 (0.59–0.63)	0.71 (0.63–0.79)	0.09 (0.04–0.13)
SMOTE	32	0.62 (0.48–0.76)	0.64 (0.62–0.66)	0.68 (0.60–0.76)	0.08 (0.04–0.12)
SMOTE	54	0.68 (0.54–0.82)	0.62 (0.60–0.64)	0.69 (0.62–0.77)	0.09 (0.05–0.13)
SMOTE	87	0.64 (0.50–0.79)	0.64 (0.62–0.66)	0.70 (0.62–0.78)	0.08 (0.04–0.13)
SMOTE+ENN	2	0.94 (0.86–1.00)	0.24 (0.23–0.26)	0.68 (0.61–0.76)	0.06 (0.03–0.08)
SMOTE+ENN	5	1.00 (1.00–1.00)	0.05 (0.04–0.06)	0.69 (0.61–0.76)	0.03 (0.03–0.04)
SMOTE+ENN	10	1.00 (1.00–1.00)	0.00 (0.00–0.00)	0.68 (0.61–0.74)	0.00 (0.00–0.00)
SMOTE+ENN	32	1.00 (1.00–1.00)	0.00 (0.00–0.00)	0.68 (0.61–0.75)	0.00 (0.00–0.00)
SMOTE+ENN	54	1.00 (1.00–1.00)	0.00 (0.00–0.00)	0.70 (0.63–0.77)	0.00 (0.00–0.00)
SMOTE+ENN	87	1.00 (1.00–1.00)	0.00 (0.00–0.00)	0.70 (0.62–0.77)	0.00 (0.00–0.00)
PCUSTe-1		0.70 (0.57–0.82)	0.59 (0.57–0.61)	0.69 (0.62–0.76)	0.08 (0.05–0.12)
PCUSTe-2		0.71 (0.58–0.83)	0.58 (0.56–0.60)	0.72 (0.64–0.79)	0.08 (0.04–0.12)

[a] Values represent point estimates on test set with 95% CIs calculated by bootstrapping test set.
[b] k: k-nearest neighbors parameter used in oversampling methods.

Table S9. Model evaluation results with hyperparameter tuning optimizing Matthews correlation coefficient (MCC) metric, using 20 most predictors selected with RFE. [a]

Model	k	Point estimate on test split (95% CI)			
		Sensitivity	Specificity	AUC	MCC
LR					
ADASYN	2	0.66 (0.53–0.79)	0.70 (0.68–0.72)	0.73 (0.66–0.80)	0.11 (0.07–0.15)
ADASYN	5	0.66 (0.53–0.79)	0.70 (0.68–0.71)	0.74 (0.67–0.80)	0.11 (0.06–0.15)
ADASYN	10	0.66 (0.53–0.79)	0.69 (0.67–0.71)	0.73 (0.66–0.81)	0.11 (0.06–0.15)
ADASYN	32	0.68 (0.55–0.81)	0.70 (0.68–0.72)	0.73 (0.66–0.81)	0.12 (0.07–0.16)
ADASYN	54	0.70 (0.57–0.83)	0.70 (0.68–0.72)	0.74 (0.67–0.81)	0.12 (0.08–0.17)
ADASYN	87	0.70 (0.57–0.83)	0.70 (0.68–0.72)	0.73 (0.66–0.81)	0.12 (0.08–0.17)
SMOTE	2	0.62 (0.49–0.75)	0.70 (0.68–0.71)	0.73 (0.66–0.80)	0.10 (0.05–0.14)
SMOTE	5	0.66 (0.53–0.80)	0.70 (0.68–0.72)	0.74 (0.67–0.81)	0.11 (0.07–0.16)
SMOTE	10	0.66 (0.53–0.79)	0.70 (0.68–0.72)	0.73 (0.66–0.80)	0.11 (0.07–0.15)
SMOTE	32	0.64 (0.50–0.78)	0.70 (0.68–0.72)	0.73 (0.66–0.80)	0.11 (0.06–0.15)
SMOTE	54	0.68 (0.55–0.82)	0.70 (0.68–0.72)	0.73 (0.66–0.80)	0.12 (0.07–0.16)
SMOTE	87	0.68 (0.55–0.81)	0.71 (0.69–0.73)	0.73 (0.66–0.80)	0.12 (0.08–0.17)
SMOTE+ENN	2	0.68 (0.56–0.81)	0.59 (0.57–0.61)	0.72 (0.65–0.79)	0.08 (0.04–0.12)
SMOTE+ENN	5	0.70 (0.57–0.84)	0.56 (0.54–0.58)	0.72 (0.65–0.79)	0.08 (0.04–0.11)
SMOTE+ENN	10	0.77 (0.66–0.89)	0.54 (0.52–0.56)	0.72 (0.65–0.78)	0.09 (0.05–0.12)
SMOTE+ENN	32	0.79 (0.68–0.91)	0.50 (0.48–0.52)	0.71 (0.64–0.78)	0.08 (0.05–0.12)
SMOTE+ENN	54	0.77 (0.65–0.89)	0.48 (0.46–0.50)	0.71 (0.64–0.78)	0.07 (0.04–0.10)
SMOTE+ENN	87	0.79 (0.68–0.91)	0.47 (0.45–0.49)	0.71 (0.63–0.78)	0.07 (0.04–0.11)
PCUSTe-1		0.68 (0.56–0.82)	0.67 (0.65–0.69)	0.74 (0.67–0.80)	0.11 (0.07–0.15)
PCUSTe-2		0.66 (0.52–0.80)	0.64 (0.62–0.66)	0.70 (0.63–0.77)	0.09 (0.05–0.13)
RF					
ADASYN	2	0.00 (0.00–0.00)	1.00 (1.00–1.00)	0.70 (0.63–0.77)	-0.00 (-0.01–0.00)
ADASYN	5	0.04 (0.00–0.11)	0.99 (0.99–1.00)	0.69 (0.61–0.76)	0.06 (-0.01–0.16)
ADASYN	10	0.15 (0.06–0.25)	0.99 (0.98–0.99)	0.69 (0.62–0.77)	0.15 (0.05–0.25)
ADASYN	32	0.09 (0.02–0.17)	0.97 (0.96–0.97)	0.67 (0.59–0.74)	0.04 (-0.01–0.10)
ADASYN	54	0.15 (0.05–0.25)	0.96 (0.95–0.97)	0.70 (0.63–0.76)	0.07 (0.01–0.14)
ADASYN	87	0.21 (0.10–0.33)	0.95 (0.94–0.96)	0.68 (0.61–0.76)	0.10 (0.03–0.17)
SMOTE	2	0.06 (0.00–0.14)	0.99 (0.98–0.99)	0.70 (0.62–0.78)	0.07 (-0.01–0.16)
SMOTE	5	0.15 (0.06–0.24)	0.97 (0.97–0.98)	0.71 (0.63–0.78)	0.10 (0.03–0.18)
SMOTE	10	0.17 (0.07–0.28)	0.96 (0.95–0.97)	0.70 (0.62–0.78)	0.09 (0.02–0.16)
SMOTE	32	0.26 (0.14–0.37)	0.95 (0.94–0.95)	0.69 (0.61–0.77)	0.12 (0.05–0.20)
SMOTE	54	0.24 (0.12–0.35)	0.93 (0.92–0.94)	0.69 (0.61–0.77)	0.09 (0.03–0.16)
SMOTE	87	0.30 (0.18–0.42)	0.92 (0.91–0.93)	0.70 (0.63–0.78)	0.12 (0.05–0.18)
SMOTE+ENN	2	0.21 (0.10–0.33)	0.96 (0.95–0.96)	0.72 (0.64–0.78)	0.11 (0.04–0.19)
SMOTE+ENN	5	0.32 (0.20–0.45)	0.91 (0.90–0.92)	0.70 (0.63–0.78)	0.11 (0.05–0.17)
SMOTE+ENN	10	0.36 (0.23–0.50)	0.85 (0.84–0.87)	0.69 (0.61–0.76)	0.09 (0.03–0.14)
SMOTE+ENN	32	0.52 (0.37–0.66)	0.77 (0.76–0.79)	0.70 (0.63–0.77)	0.10 (0.05–0.14)
SMOTE+ENN	54	0.52 (0.37–0.65)	0.74 (0.72–0.75)	0.68 (0.61–0.75)	0.08 (0.03–0.13)
SMOTE+ENN	87	0.56 (0.41–0.70)	0.71 (0.70–0.73)	0.69 (0.61–0.76)	0.09 (0.04–0.13)
PCUSTe-1		0.66 (0.52–0.79)	0.65 (0.63–0.67)	0.70 (0.63–0.76)	0.09 (0.05–0.13)
PCUSTe-2		0.73 (0.59–0.85)	0.68 (0.66–0.70)	0.75 (0.68–0.81)	0.12 (0.07–0.17)

SGD						
	ADASYN	2	0.64 (0.51–0.78)	0.68 (0.66–0.70)	0.72 (0.65–0.78)	0.10 (0.05–0.14)
	ADASYN	5	0.62 (0.49–0.76)	0.68 (0.66–0.71)	0.72 (0.65–0.79)	0.09 (0.05–0.14)
	ADASYN	10	0.66 (0.53–0.79)	0.69 (0.67–0.70)	0.73 (0.66–0.79)	0.11 (0.06–0.15)
	ADASYN	32	0.64 (0.50–0.77)	0.69 (0.67–0.71)	0.72 (0.66–0.79)	0.10 (0.06–0.14)
	ADASYN	54	0.62 (0.48–0.75)	0.69 (0.67–0.71)	0.72 (0.66–0.79)	0.09 (0.05–0.14)
	ADASYN	87	0.62 (0.48–0.75)	0.68 (0.67–0.70)	0.72 (0.65–0.79)	0.09 (0.05–0.14)
	SMOTE	2	0.73 (0.60–0.85)	0.70 (0.68–0.72)	0.74 (0.67–0.81)	0.13 (0.09–0.17)
	SMOTE	5	0.73 (0.60–0.85)	0.70 (0.68–0.72)	0.75 (0.68–0.82)	0.13 (0.09–0.17)
	SMOTE	10	0.73 (0.60–0.85)	0.70 (0.69–0.72)	0.74 (0.67–0.81)	0.13 (0.09–0.17)
	SMOTE	32	0.73 (0.60–0.86)	0.71 (0.69–0.73)	0.74 (0.67–0.81)	0.13 (0.09–0.18)
	SMOTE	54	0.70 (0.58–0.84)	0.71 (0.69–0.73)	0.74 (0.67–0.81)	0.13 (0.09–0.17)
	SMOTE	87	0.73 (0.60–0.86)	0.71 (0.69–0.72)	0.75 (0.68–0.82)	0.13 (0.09–0.18)
	SMOTE+ENN	2	0.73 (0.60–0.85)	0.62 (0.60–0.64)	0.73 (0.67–0.79)	0.10 (0.06–0.14)
	SMOTE+ENN	5	0.71 (0.57–0.83)	0.58 (0.56–0.60)	0.73 (0.67–0.80)	0.08 (0.04–0.12)
	SMOTE+ENN	10	0.75 (0.63–0.87)	0.54 (0.52–0.56)	0.73 (0.66–0.79)	0.08 (0.05–0.12)
	SMOTE+ENN	32	0.79 (0.68–0.91)	0.50 (0.48–0.52)	0.73 (0.66–0.79)	0.08 (0.05–0.12)
	SMOTE+ENN	54	0.79 (0.67–0.91)	0.48 (0.45–0.50)	0.72 (0.66–0.78)	0.08 (0.04–0.11)
	SMOTE+ENN	87	0.81 (0.70–0.91)	0.46 (0.44–0.48)	0.72 (0.65–0.78)	0.08 (0.04–0.11)
	PCUSTe-1		0.60 (0.46–0.74)	0.66 (0.65–0.68)	0.69 (0.62–0.76)	0.08 (0.03–0.12)
	PCUSTe-2		0.64 (0.51–0.78)	0.70 (0.68–0.72)	0.72 (0.65–0.79)	0.10 (0.06–0.15)
SVM						
	ADASYN	2	0.60 (0.46–0.74)	0.67 (0.65–0.69)	0.70 (0.62–0.77)	0.08 (0.04–0.12)
	ADASYN	5	0.64 (0.50–0.78)	0.67 (0.65–0.69)	0.71 (0.63–0.77)	0.09 (0.05–0.14)
	ADASYN	10	0.62 (0.48–0.75)	0.67 (0.65–0.69)	0.70 (0.63–0.77)	0.09 (0.04–0.13)
	ADASYN	32	0.60 (0.45–0.74)	0.68 (0.66–0.69)	0.70 (0.62–0.77)	0.08 (0.04–0.13)
	ADASYN	54	0.62 (0.47–0.75)	0.68 (0.66–0.69)	0.70 (0.62–0.77)	0.09 (0.05–0.13)
	ADASYN	87	0.62 (0.47–0.75)	0.67 (0.65–0.69)	0.69 (0.62–0.77)	0.09 (0.04–0.13)
	SMOTE	2	0.62 (0.47–0.76)	0.67 (0.65–0.69)	0.70 (0.62–0.77)	0.09 (0.04–0.13)
	SMOTE	5	0.60 (0.45–0.73)	0.67 (0.65–0.69)	0.71 (0.63–0.78)	0.08 (0.04–0.12)
	SMOTE	10	0.60 (0.45–0.73)	0.67 (0.65–0.69)	0.70 (0.62–0.77)	0.08 (0.03–0.12)
	SMOTE	32	0.64 (0.50–0.78)	0.68 (0.66–0.70)	0.70 (0.62–0.77)	0.10 (0.05–0.14)
	SMOTE	54	0.60 (0.44–0.74)	0.67 (0.65–0.69)	0.70 (0.62–0.77)	0.08 (0.04–0.13)
	SMOTE	87	0.62 (0.47–0.75)	0.68 (0.66–0.69)	0.70 (0.62–0.77)	0.09 (0.04–0.13)
	SMOTE+ENN	2	0.73 (0.60–0.85)	0.62 (0.60–0.64)	0.74 (0.68–0.80)	0.10 (0.06–0.14)
	SMOTE+ENN	5	0.73 (0.60–0.85)	0.58 (0.56–0.60)	0.74 (0.68–0.80)	0.09 (0.05–0.13)
	SMOTE+ENN	10	0.75 (0.63–0.87)	0.55 (0.53–0.57)	0.73 (0.67–0.79)	0.08 (0.05–0.12)
	SMOTE+ENN	32	0.83 (0.71–0.94)	0.51 (0.49–0.53)	0.73 (0.67–0.79)	0.10 (0.06–0.13)
	SMOTE+ENN	54	0.81 (0.70–0.92)	0.48 (0.46–0.50)	0.72 (0.66–0.79)	0.08 (0.05–0.12)
	SMOTE+ENN	87	0.79 (0.68–0.91)	0.47 (0.45–0.49)	0.72 (0.66–0.79)	0.07 (0.04–0.11)
	PCUSTe-1		0.64 (0.50–0.78)	0.66 (0.64–0.68)	0.72 (0.65–0.79)	0.09 (0.05–0.13)
	PCUSTe-2		0.62 (0.49–0.76)	0.67 (0.65–0.69)	0.71 (0.63–0.79)	0.09 (0.04–0.13)
XGBoost						
	ADASYN	2	0.45 (0.31–0.59)	0.82 (0.80–0.83)	0.71 (0.64–0.78)	0.10 (0.04–0.15)
	ADASYN	5	0.47 (0.33–0.61)	0.80 (0.79–0.82)	0.72 (0.65–0.79)	0.10 (0.05–0.15)
	ADASYN	10	0.45 (0.31–0.60)	0.80 (0.79–0.82)	0.72 (0.65–0.78)	0.09 (0.04–0.14)

ADASYN	32	0.49 (0.36–0.63)	0.80 (0.79–0.82)	0.71 (0.64–0.78)	0.10 (0.06–0.15)
ADASYN	54	0.45 (0.31–0.60)	0.81 (0.79–0.82)	0.72 (0.65–0.79)	0.09 (0.04–0.15)
ADASYN	87	0.47 (0.33–0.62)	0.80 (0.79–0.82)	0.71 (0.64–0.78)	0.10 (0.05–0.15)
SMOTE	2	0.30 (0.18–0.43)	0.92 (0.91–0.93)	0.70 (0.62–0.77)	0.11 (0.05–0.18)
SMOTE	5	0.36 (0.23–0.49)	0.91 (0.89–0.92)	0.70 (0.63–0.78)	0.13 (0.06–0.19)
SMOTE	10	0.32 (0.20–0.45)	0.90 (0.89–0.92)	0.70 (0.63–0.77)	0.11 (0.04–0.16)
SMOTE	32	0.28 (0.16–0.41)	0.90 (0.89–0.91)	0.70 (0.62–0.77)	0.08 (0.03–0.15)
SMOTE	54	0.30 (0.18–0.43)	0.89 (0.88–0.90)	0.70 (0.62–0.77)	0.09 (0.03–0.14)
SMOTE	87	0.30 (0.18–0.43)	0.89 (0.88–0.90)	0.70 (0.63–0.77)	0.09 (0.03–0.14)
SMOTE+ENN	2	0.39 (0.25–0.52)	0.84 (0.83–0.86)	0.72 (0.66–0.79)	0.09 (0.04–0.14)
SMOTE+ENN	5	0.47 (0.33–0.61)	0.80 (0.79–0.82)	0.72 (0.65–0.78)	0.10 (0.05–0.15)
SMOTE+ENN	10	0.49 (0.35–0.63)	0.76 (0.74–0.78)	0.71 (0.64–0.78)	0.08 (0.04–0.13)
SMOTE+ENN	32	0.64 (0.51–0.77)	0.71 (0.69–0.73)	0.71 (0.64–0.78)	0.11 (0.06–0.15)
SMOTE+ENN	54	0.62 (0.48–0.75)	0.69 (0.67–0.70)	0.72 (0.64–0.78)	0.09 (0.05–0.13)
SMOTE+ENN	87	0.64 (0.50–0.78)	0.68 (0.66–0.69)	0.72 (0.65–0.79)	0.10 (0.05–0.14)
PCUSTe-1		0.66 (0.53–0.80)	0.65 (0.63–0.67)	0.71 (0.65–0.78)	0.09 (0.05–0.13)
PCUSTe-2		0.66 (0.53–0.79)	0.62 (0.60–0.64)	0.68 (0.61–0.76)	0.08 (0.04–0.12)
Inc. XGBoost					
ADASYN	2	0.62 (0.48–0.75)	0.66 (0.64–0.67)	0.68 (0.61–0.75)	0.08 (0.04–0.12)
ADASYN	5	0.68 (0.55–0.82)	0.62 (0.60–0.64)	0.70 (0.63–0.78)	0.09 (0.05–0.13)
ADASYN	10	0.64 (0.50–0.77)	0.62 (0.60–0.63)	0.71 (0.64–0.78)	0.07 (0.03–0.11)
ADASYN	32	0.64 (0.51–0.78)	0.62 (0.60–0.64)	0.68 (0.61–0.76)	0.08 (0.04–0.12)
ADASYN	54	0.64 (0.51–0.78)	0.64 (0.62–0.66)	0.70 (0.64–0.78)	0.08 (0.04–0.12)
ADASYN	87	0.62 (0.48–0.76)	0.66 (0.64–0.68)	0.71 (0.65–0.78)	0.08 (0.04–0.13)
SMOTE	2	0.60 (0.45–0.74)	0.72 (0.70–0.74)	0.68 (0.61–0.75)	0.10 (0.05–0.15)
SMOTE	5	0.60 (0.46–0.74)	0.72 (0.70–0.73)	0.70 (0.63–0.77)	0.10 (0.05–0.14)
SMOTE	10	0.57 (0.44–0.71)	0.73 (0.71–0.75)	0.70 (0.63–0.77)	0.10 (0.05–0.14)
SMOTE	32	0.58 (0.44–0.71)	0.72 (0.70–0.74)	0.72 (0.64–0.78)	0.09 (0.05–0.14)
SMOTE	54	0.58 (0.44–0.72)	0.74 (0.72–0.75)	0.72 (0.66–0.78)	0.10 (0.05–0.15)
SMOTE	87	0.53 (0.40–0.67)	0.73 (0.72–0.75)	0.71 (0.64–0.77)	0.09 (0.04–0.13)
SMOTE+ENN	2	0.83 (0.72–0.94)	0.40 (0.38–0.42)	0.70 (0.63–0.76)	0.07 (0.03–0.10)
SMOTE+ENN	5	0.96 (0.89–1.00)	0.23 (0.21–0.24)	0.72 (0.64–0.79)	0.06 (0.04–0.08)
SMOTE+ENN	10	1.00 (1.00–1.00)	0.00 (0.00–0.00)	0.69 (0.63–0.76)	0.00 (0.00–0.00)
SMOTE+ENN	32	1.00 (1.00–1.00)	0.00 (0.00–0.00)	0.68 (0.61–0.75)	0.00 (0.00–0.00)
SMOTE+ENN	54	1.00 (1.00–1.00)	0.00 (0.00–0.00)	0.71 (0.64–0.77)	0.00 (0.00–0.00)
SMOTE+ENN	87	1.00 (1.00–1.00)	0.00 (0.00–0.00)	0.69 (0.62–0.76)	0.00 (0.00–0.00)
PCUSTe-1		0.66 (0.53–0.80)	0.61 (0.59–0.63)	0.70 (0.63–0.77)	0.08 (0.04–0.12)
PCUSTe-2		0.60 (0.45–0.73)	0.63 (0.61–0.65)	0.66 (0.57–0.74)	0.07 (0.02–0.11)

[a] Values represent point estimates on test set with 95% CIs calculated by bootstrapping test set.
[b] k: k-nearest neighbors parameter used in oversampling methods.

Table S10. Secondary performance metrics of the best performing models. [a]

Accuracy (95% CI)	PPV [b] (95% CI)	NPV [c] (95% CI)	Brier score (95% CI)	F1 score (95% CI)
Null model [d]				
0.02	0.02	0.00	0.02	0.00
LR				
0.71 (0.69–0.72)	0.05 (0.03–0.06)	0.99 (0.99–1.00)	0.02 (0.01–0.03)	0.81 (0.79–0.82)
SGD				
0.65 (0.63–0.67)	0.04 (0.03–0.06)	0.99 (0.99–1.00)	0.02 (0.02–0.03)	0.77 (0.75–0.78)
RF				
0.62 (0.60–0.64)	0.04 (0.03–0.05)	0.99 (0.99–1.00)	0.02 (0.01–0.03)	0.75 (0.73–0.76)
XGBoost				
0.61 (0.59–0.63)	0.04 (0.03–0.05)	0.99 (0.99–1.00)	0.02 (0.02–0.03)	0.74 (0.72–0.75)
Inc. XGBoost				
0.62 (0.60–0.64)	0.04 (0.03–0.05)	0.99 (0.98–0.99)	0.02 (0.02–0.03)	0.75 (0.73–0.76)
SVM				
0.60 (0.58–0.62)	0.04 (0.03–0.05)	0.99 (0.99–1.00)	0.02 (0.01–0.03)	0.73 (0.72–0.75)

[a] Values represent point estimates on test set with 95% CIs calculated by bootstrapping.
[b] PPV: positive predictive value.
[c] NPV: negative predictive value.
[d] Null model was defined as incidence-only model that always predicts positive class.

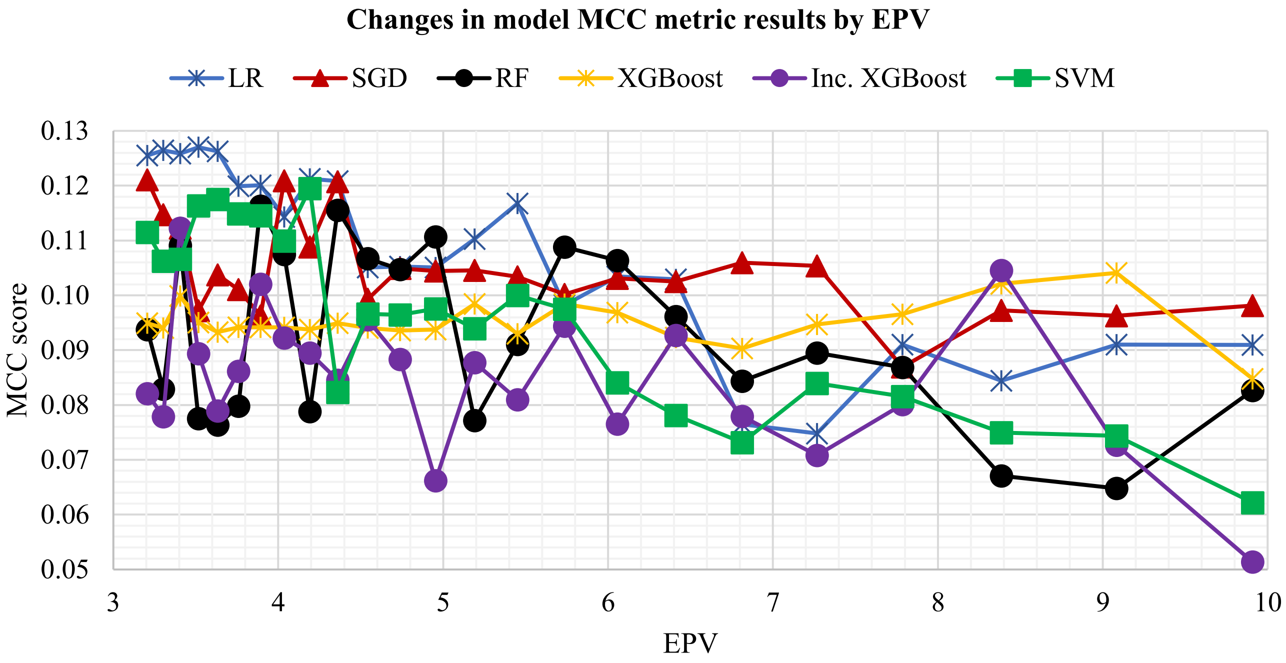

Figure S2. Matthews correlation coefficient (MCC) at relative event-per-variable (EPV) values (11 to 34 predictors).

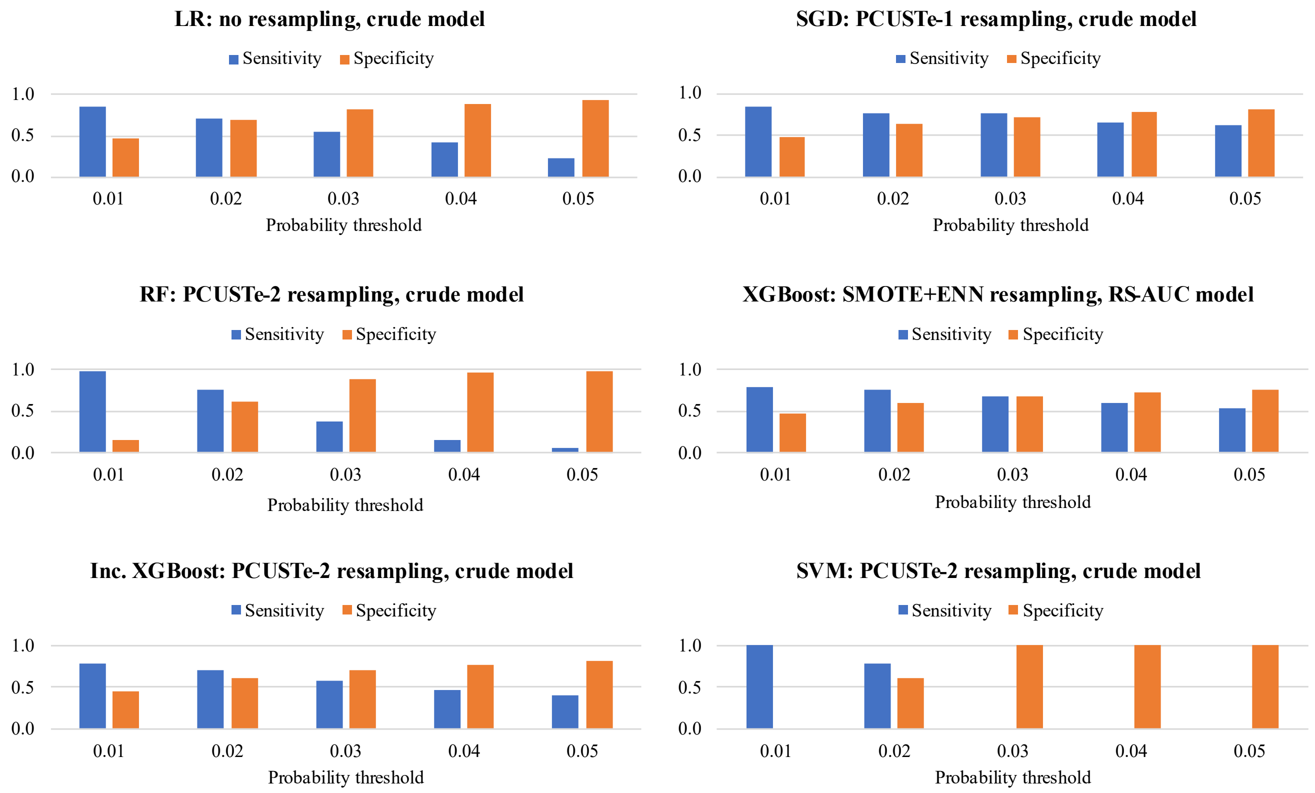

Figure S3. Sensitivity and specificity metrics at relative decision thresholds (1~5% disease prevalence).

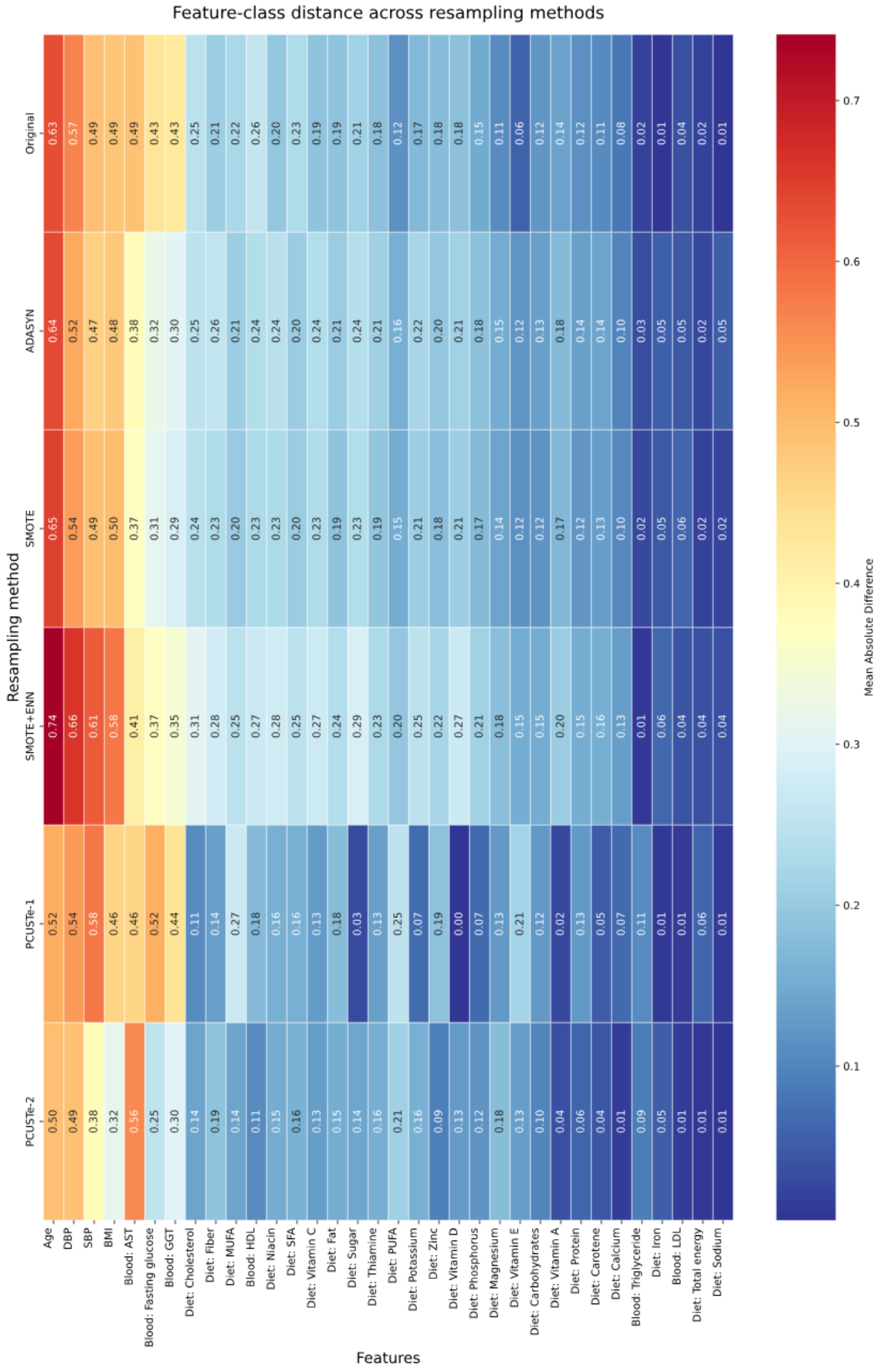

Figure S4. Predictor-wise mean absolute differences between cases and controls for each resampled and original train dataset. All predictors were scaled prior this comparison using adapted Z-score normalization.
